# Supplementary material for: Watching subtitled videos with the sound off affects viewers’ comprehension, cognitive load, immersion, enjoyment, and gaze patterns: A mixed-methods eye-tracking study
Source: PLoS One. 2024 Oct 7;19(10):e0306251. doi: 10.1371/journal.pone.0306251 (PMC11458047; doi:10.1371/journal.pone.0306251)
Supplement: Attachment — Submitted filename: Response to reviewers.pdf [file pone.0306251.s003.pdf]

Response to Reviewers

Manuscript: Watching subtitled videos with the sound off affects viewers' comprehension, cognitive load, immersion, enjoyment, and gaze patterns: A mixed methods eye-tracking study

First, we would like to start by expressing our gratitude to the Reviewers for their examination of our manuscript and for the valuable feedback they provided. We trust that their suggestions have contributed to enhancing our manuscript.

All changes can be located in the attached word file with tracked changes. We also attached a clean version, with all changes accepted. Below, we address specific issues raised by the Editors first and then by each reviewer.

All reviewers' and editors' comments are marked in blue. The changes we introduced in the manuscript are reported in green.

Editors

1. Please ensure that your manuscript meets PLOS ONE's style requirements, including those for file naming. The PLOS ONE style templates can be found at https://journals.plos.org/plosone/s/file?id=wjVg/PLOSOne_formatting_sample_main_body.pdf and https://journals.plos.org/plosone/s/file?id=ba62/PLOSOne_formatting_sample_title_authors_affiliations.pdf

We have gone through the file naming conventions and confirm that they manuscript meets the requirements.

[Comment 2 disregarded, following the correspondence with Teresa Diviacchi, Publishing Editor, on 21 February 2024].

3. Thank you for stating the following financial disclosure:
"This study was conducted within the WATCH-ME project funded by the National Science Centre OPUS 19 programme 2020/37/B/HS2/00304. The principal investigator is Agnieszka Szarkowska, whereas Valentina Ragni is employed as a post-doc and Sonia Szkriba as a PhD candidate researcher in the project."

Please state what role the funders took in the study. If the funders had no role, please state: "The funders had no role in study design, data collection and analysis, decision to publish, or preparation of the manuscript."
If this statement is not correct you must amend it as needed.
Please include this amended Role of Funder statement in your cover letter; we will change the online submission form on your behalf.

The funder did not have any role in the study, so we confirm that this statement is true: "The funders had no role in study design, data collection and analysis, decision to publish, or preparation of the manuscript."

4. Please include your full ethics statement in the 'Methods' section of your manuscript file. In your statement, please include the full name of the IRB or ethics committee who approved or waived your study, as well as whether or not you obtained informed written or verbal consent. If consent was waived for your study, please include this information in your statement as well.

We have included the full ethics statement in the first version of the manuscript we submitted, including the full names of the ethics committees and the fact that the consent was written. Here is the relevant fragment:

Ethics clearance was secured from the ethics committees of each institution where data collection took place (**Rector's Committee for the Ethics of Research Involving Human Participants at the University of Warsaw, Faculty of Arts and Humanities Research Ethics Subcommittee at the University of East Anglia, and Human Sciences Subcommittee at Macquarie University**). Upon arrival at the laboratory, participants were provided with an information sheet about the study and had the opportunity to ask questions. After reviewing the information, participants proceeded to sign a **written** consent form in order to participate in the study.

All the documentation related to ethics, including the clearances and their reference numbers, have been submitted together with the manuscript.

5. Thank you for considering our journal for publishing your research. Your manuscript has now been peer reviewed and I agree with comments provided, particularly with those provided by reviewers number 2 and 3. I consider that your methods and analysis should be properly enhanced so we can consider it for publication. Could I ask authors to comprehensively review

these aspects of your manuscript and provide a new re-analysis of your data following the reviewers recommendations, please?

Thank you for considering our manuscript for publication in PLoS ONE. We appreciate all the comments and suggestions, and we address each of them below.

As requested, we have re-run our analyses to include gender, age, and years of education. We have also calculated Cronsbach alpha to validate the internal consistency of the cognitive load instrument we used. We report the details below.

Reviewer 1

- Overall, this is a good paper about Watching subtitled videos with the sound off affects viewers' comprehension, cognitive load, immersion, enjoyment, and gaze patterns.

- The introduction and presentation of the problem do not properly fulfil their roles: they do not introduce or appropriately address the issues of the research. The research problem is not clear, and it needs to be supported and explained why the researchers did the research.

We have rewritten the Introduction to better present the research problem and added a section on Theoretical Framework (see below).

- How general are your results? These have to be of interest to the whole community؛

We believe our results are relevant to viewers across the globe, who increasingly engage in watching videos, especially on social media, with the sound turned off. This global trend, confirmed by a number of publications coming from different countries (some of which we list below), calls for research on how the new watching habits affect viewers.

Kanter, J., *Young people use subtitles to get the plot*, in *The Times*. 2021. p. 20.
Pulse *The Majority of Young People Are Using Subtitles When They Watch TV*. YPulse, 2022.
Zajechowski, M., *Survey: Why America is obsessed with subtitles*. 2022, Preply.
Forsberg, C. *Subtitles become popular among general population due to changes in TV, film consumption*. ABC News: Australian Broadcasting Corporation., 2023.
Cunningham, K., *Mumbling actors, bad speakers or lazy listeners? Why everyone is watching TV with subtitles on*, in *The Guardian*. 2023.
McCue, T., *Verizon Media Says 69 Percent Of Consumers Watching Video With Sound Off*, in *Forbes*. 2019.

Having conducted a study on two continents among a large number of participants, and having analysed the data with inferential statistics, whose role is to allow us to extrapolate our findings to other populations, extending beyond our research sample, we believe that the results are of interest to the community - both and the general public and researchers,

particularly from the fields such as Translation Studies, Audiovisual Translation, Applied Linguistics, Psycholinguistics, Cognitive Psychology and Psycholinguistics.

- Interpretations of the results should be provided in light of previous studies and training theories;

Thank you for this comment. We have added more studies and theories to the introductory part of the manuscript, in particular Schnotz (2005) and Mayer (2005 and 2014). Please see the next point for details.

- No relevant theories were included in the discussion to support or provide anchorage to the research problem.

We added a section "Theoretical framework" in the introductory part of the manuscript. We hope it provides support to the research problem presented. We also added references to the theories in the Discussion.

Theoretical framework

Watching subtitled video requires the coordination of complex mental processes that have not been explored extensively in the literature. There are some theoretical models and frameworks to explain the integration of text, image and sound, such as the integrated model of text and picture comprehension [16]; the multimodal integrated-language framework [17, 18]; and the cognitive theory of multimedia learning [19, 20]. The framework by Liao at al. [17] investigates this integration in the context of dynamic texts like video with subtitles and a soundtrack. Both the model by Schnotz [16] and Mayer's theory [20] understand the cognitive architecture as based on input from auditory and visual sensory modalities and a limited working memory capacity. They highlight how split attention in contexts like watching subtitled video without sound could impede comprehension. In Schnotz's model [16], split attention would result in both descriptive processing (of words) and depictive processing (of sounds and images) being dependent on visual perception, thereby increasing cognitive load. In Mayer's theory [20], the multimedia principle holds that a combination of words and pictures are better for learning than words alone, whereas the modality principle assumes that presenting information in two modalities is better for comprehension than presenting information only in one modality. This theory, like

Schnotz's model, would imply that subtitled video without sound would therefore increase cognitive load.

Neither Schnotz's model nor Mayer's theory, however, specify how the processing of one source of information might impact on another (e.g., video and subtitles). The multimodal integrated-language framework of Liao et al. [17, 18] builds on models of eye movement control during reading to predict how the reading of subtitles might be impacted by the concurrent presence of visual and auditory information. The framework assumes that reading is a serial process (i.e., sentence processing is contingent on the strictly sequential identification of words) and that objects in a scene likewise have to be fixated sequentially for identification due to the limitations of visual perception. However, the auditory processing of speech and sounds and the tracking of previously identified objects can occur in parallel with these serial processes. In the absence of sound, the benefit of parallel processing of sound disappears, forcing the viewer to rely on the sequential processing of image and subtitles.

This article will attempt to confirm some of the findings of Liao et al. [17], by exploring whether greater reliance on subtitles in the absence of sound will result in more time on the subtitles, longer fixations, and shorter saccades (or lower word skipping), reflecting the prioritization of the subtitles when the benefit of parallel processing of sound is absent.

Many thanks to the study team for the remarkable research they conducted and the unique manner in which it was presented.
Thank you! We appreciate your positive feedback on our work.

Reviewer 2

1.The paper frequently employs long sentences, and breaking some of them would improve overall comprehension.

Thank you for this comment. Following your suggestion, we have revised the manuscript and shortened long sentences to improve readability.

2.Elaborate on how the sample size was estimated (justification).

When designing the study and calculating the required minimum number of participants, we followed the suggestions put forward by Brysbaert (2019):

- We used a within-subject (rather than between-subject) design to increase power.
- In the eye-tracking part, we used multiple observations per condition per participant (each subtitle in each clip was used as a data point in each of the two conditions for each participant) in mixed models analyses.
- For the ANOVA test, a minimum of 27 participants per condition in a two-way ANOVA with one repeated-measures factor is recommended, assuming the power of 80% and a medium effect size.
- We aimed to recruit at least 40 participants per group on the assumption that some data will have to be discarded due to poor quality, as is normally the case in any eye-tracking study.

Source:

Brysbaert, M. (2019). How Many Participants Do We Have to Include in Properly Powered Experiments? A Tutorial of Power Analysis with Reference Tables. Journal of Cognition, 2(1), 16. https://doi.org/10.5334/joc.72

3.Sampling technique employed (how) and related sampling bias should be discussed.

We used convenience and snowball sampling methods. Participants were recruited based on the criteria of being either a Polish or English native speaker, between the ages of 18 and 55. We used the pools of participants available at the institutions taking part in the study, social media, notice boards, and personal contacts to recruit participants.

At Macquarie University, we could only use people on campus, and as the campus is rather distant from the city, we effectively tested students. This resulted in a skewed sample of Australian participants, and a resulting confound with age: as we acknowledge in the manuscript, age was confounded with the participants provenance.

4.The justification of why different apparatuses were used for eye movement measurements, as well as differing distances from the screen and potential biases, should be elaborated on.

Many thanks for asking an important question. We used an EyeLink Plus 1000 (University of Warsaw, Poland; Macquarie University, Sydney, Australia) and a Portable Duo (University of East Anglia, UK) because these are the systems the research group had in place in the different locations of data collection. Prior to data collection, we enquired precisely about this with the manufacturer (SR Research, Canada), who confirmed that they have done extensive testing to determine the specifications of both systems, and the results show that data quality is comparable for the two systems.

The technical characteristics of both systems are provided below.

Eyelink 1000 Plus: https://www.sr-research.com/wp-content/uploads/2017/11/eyelink-1000-plus-specifications.pdf
Eyelink Portable Duo: https://www.sr-research.com/wp-content/uploads/2017/07/portable-duo-specifications.pdf

First of all, the technical characteristics of both systems are similar and fully comparable. Second, the data file format is the same across the two systems. Third, the systems use the same underlying technology, including the same eye tracking algorithms. Fourth, experiment creation procedures (through the Experiment Builder software) and data analysis options (in the Data Viewer software) are the same. We ran exactly the same Experiment Builder project and used exactly the same data analysis scripts for both setups.

The differences across the systems (e.g., cable types, mounting options) should not affect data quality, data collection, or data analysis pipelines. The reviewers can find a side-by-side comparison of specs from the manufacturer here, where these differences are described: https://www.sr-research.com/wp-content/uploads/2021/03/EyeLink-Portable-Duo-and-1000-Plus-Specs.pdf

Key experimental elements that could introduce discrepancies and therefore confounds are (a) the sampling frequency used during data collection, (b) presentation rates of the videos, (c) refresh rates of the monitor screen and (d) the average accuracy of the machines. We made sure that (a) all data was recorded at 1000 Hz in both systems, (b) presentation rates were kept the same in all videos in both setups (25 fps, frames per second), (c) monitor refresh rates were the same (60 Hz) and (d) the accuracy specs of both systems is also the same (typically 0.25° to 0.5°, as you can see from the links above).

Lastly, as for the different eye-to-screen distances, these are determined by the size and position of the monitor screen. A different distance is not synonymous with incompatibility but is in fact required for letters to subtend a comparable portion of the visual angle in both setups. Because the Portable Duo is mounted on a specialised laptop (provided by the manufacturer) with predetermined screen dimensions, whereas the 1000 Plus is used with a separate monitor screen, in order for the participants to see the same size subtitle letters, sitting at different distances is necessary. As we stated in the method section, in both setups each subtitle letter subtended ~0.32° of the visual angle, which ensured comparability and was achieved by finding the ideal distance between the participant and two different screens.

We hope that the detailed explanation above clarifies why the systems can be used in the same study without affecting data quality of comparability. In the manuscript, we added the following information for clarity:

The different optimised participant-to-screen distances ensured comparability, such that in all experimental setups, each subtitle letter subtended ~0.32° of the visual angle.

5. Highlight on the study duration and period it was carried out.
We added this information to the manuscript in the Method section:

The data collection started in Poland on 29 November 2022 and finished in Australia on 26 May 2023.

6. The study topic under study could be influenced or differ by sex or gender. Any sex-related analysis in any of the analyses performed could have provided deeper insights. Authors should justify why such related analyses were not considered as well as why they did not consider controlling for "any variable" that potentially may have influenced the relations (for instance, how are we sure the differences observed are not more due to age, education background, familiarity with technology, or subtitles other than absence of sound?).

We did not run the analyses on gender to begin with because it was not theoretically motivated and was not part of our research questions. Gender was not reported as a significant variable in any previous eye-tracking study on subtitling, so there has been no literature to prompt us to investigate this. Moreover, our research design in the questionnaire part already has two independent variables, so adding another factor would reduce the power. Further, the gender analysis is complicated by the fact that our participants declared to be not only male or female, but also non-binary (4 people). As we didn't have more non-binary people, we would have to exclude them from the analyses to avoid the data being severely skewed.

However, in spite of the above explanations, we followed the Reviewer's suggestion and we re-ran ANOVAs on Comprehension, Recall, Immersion, Enjoyment and Cognitive Load with gender as a factor. None of them found any significant differences between the genders.

Regarding the educational background, we collected information about the number of years of education they completed. This variable naturally correlated with participants' age. Age, in turn, was not added to the model (ANCOVA), as it did not meet the preliminary assumptions: there were no correlations of age with any dependent variables.

To address the reviewers' comments under points 6 and 7, we also re-ran our eye-tracking analyses. We re-ran each of the four final mixed-effect regression models (TRT, MFD, CC, SKIP) adding the following predictor variables as covariates: gender, age, education (years of instruction), and familiarity with subtitles. Please see the end of each analysis file (provided both as .html and .Rmd), under section "Covariates addition". Overall, adding these predictors (including age) did not consistently improve model fit nor substantially change model results, though with some exceptions. Below we provide details of each eye-movement analysis.

In the **TRT** analysis, none of the four additional predictors contributed to improve model fit. Gender did not have a significant effect on TRT (M: $b = -14.632$, $SE = 41.504$, $t = -0.353$, $p = 0.7249$; NB: $b = 14.665$, $SE = 100.174$, $t = 0.146$, $p = 0.8838$), neither did education ($b = -9.836$, $SE = 7.433$, $t = -1.323$, $p = 0.1877$), subtitling preference ($b = -21.703$, $SE = 53.663$, $t = -0.404$, $p = 0.6865$), or age ($b = 5.369$, $SE = 2.968$, $t = 1.809$, $p = 0.0724$). In the gender analysis, there are two coefficients because there were three genders in total, as alongside M and F (baseline reference level), some participants identified as non-binary (NB). Just like in the original analysis, the only significant predictors of TRT remained language group (AUS/PL/UK) and sound condition (ON/OFF). The original final model with only our two key predictors of interest

(language group, sound condition) explained a 75.2% of the total variance in TRT (Conditional R2 = 0.752), whereas the full model with all covariates included explains 75.3% variance (Conditional R2 = 0.753), i.e., a nearly identical amount. Considering that the R2 naturally grows as more variables are added to the model, the fact that the addition of as many as four predictor variables barely increased the R2 at all, clearly indicates that these four variables had no systematic effect on TRT.

In the **MFD** analysis, none of the four additional predictors contributed to improve model fit. Gender did not have a significant effect on TRT (M: b = -0.003023, SE = 0.02395, t = -0.126, p = 0.899; NB: b = -0.05042, SE = 0.05779, t = -0.872, p = 0.384), neither did education (b = -0.0009366, SE = 0.004288, t = -0.218, p = 0.827), subtitling preference (b = -0.01371, SE = 0.03097, t = -0.443, p = 0.658), or age (b = -0.002844, SE = 0.001712, t = -1.661, p = 0.098. Just like in the original analysis, the only significant predictor of MFD was the sound condition (ON/OFF). The original final model with only our two key predictors of interest explained 30.4% of the total variance in MFD (Conditional R^2 = 0. 304), whereas the full model with all covariates included explains 30.9% variance (Conditional R^2 = 0. 309), which is a negligible increase. Considering that the R^2 naturally grows as more variables are added to the model, the fact that the addition of as many as four predictor variables increased the R^2 by a minimal amount clearly indicates that these four variables had no systematic effect on MFD.

In the **CC** analysis, none of the four additional predictors contributed to improve model fit. Gender did not have a significant effect on CC (M: b = 0.029369, SE = 0.035202, t = 0.834, p = 0.405; NB: b = 0.054705, SE = 0.084586, t = 0.647, p = 0.518), neither did education (b = -0.003450, SE = 0.006295, t = -0.548, p = 0.584), subtitling preference (b = -0.056164, SE = 0.045384, t = -1.238, p = 0.2180), or age (b = -0.003012, SE = 0.002515, t = -1.197, p = 0.233). Unlike for TRT and MFD, adding these predictors changed the original CC analysis results. In the original analysis, the only significant predictor of CC was language group (AUS cohort). In this latest additional analysis with all covariates, the AUS group is no longer significant (b = 0.081321, SE = 0.042266, t = 1.924, p = 0.056), particularly after Bonferroni correction for multiple comparisons, whereby only p values under 0.0125 (0.05/4) should be considered significant. Since model results changed, we compared the original model (‘cc.lmer.17’) to the additional one (‘cc.lmer.17.extra’) to assess which model provides a better fit to the data. The results showed that the original model has both lower AIC and BIC, indicating that adding the four extra predictors does not provide a better fit. Moreover, the original final model with only our two key predictors of interest explained 23.8% of the total variance in CC (Conditional R^2 = 0.238), whereas the full model with all covariates included explains a nearly identical amount (Conditional R^2 = 0.239). Comparisons of both R^2 and AIC/BIC therefore confirm that the four extra predictors had no sizable effect on CC and their addition does not add substantial explanatory power to the model.

In the **skipping** analysis, subtitling preference did not have a significant effect on skipping probability (b = 0.22830, SE = 0.29743, z = 0.768, p = 0.442), whereas education and age did. Specifically, the probability of skipping was higher for those with higher education (no. of years studied) (b = 0.10731, SE = 0.04114, z = 2.608, p = 0.009) and lower age (b = -0.06197, SE = 0.01702, z = -3.640, p < 0.001). gender also did not have any significant effect on skipping: both male (b = 0.09447, SE = 0.22843, t = 0.414, p = 0.679) and non-binary viewers (b = -

1.09458, SE = 0.55056, z = -1.988, p = 0.046) skipped subtitles in comparable amounts to the baseline female group. The non-binary gender coefficient is not significant despite the p value of 0.04 because of the Bonferroni correction for multiple comparisons. Importantly, just like in the original analysis, language group (AUS/PL/UK) and sound condition (ON/OFF) remained significant skipping predictors, with the L2-English (L1-Polish) cohort being significantly less likely to skip subtitles.

Since in this analysis some of the covariates did appear to have an effect, we compared the original model ('s.glmer.skip.26') to the additional one ('s.glmer.skip.26.extra.1') to assess which model provides a better fit to the data. The new model has a lower AIC but higher BIC than the original one, so it does not immediately and incontrovertibly emerge as a better fit to the data. Moreover, the original final model with only our two key predictors of interest explained 48.3% of the total variance in TRT (Conditional $R^2 = 0.483$), while the full model with all covariates included explains an identical amount (Conditional $R^2 = 0.483$), which also points towards the overall questionability of the full model with all covariates. It must also be noted that adding four predictors to the original model (which already had three predictors, namely language group, the presence/absence of sound, and their interaction) takes up degrees of freedom and will decrease power.

In summary, out of the four covariates added to the models in this latest round of re-analysis, subtitling preference and gender never had any effect on any of the eye-movement measures analysed, whereas education and age only affected skipping. More importantly, the patterns of significance remained largely the same for all eye-movement measures, confirming the original results we report and discuss in the paper. The only exception was crossover counts (CC), where the original AUS cohort effect disappeared – however, this effect was unexpected in the first place (as we elaborate on in the manuscript's Discussion) and, if anything, this result confirms that said unexpected effect is weak and does not emerge consistently, which is well-aligned with our overall expectations.

We did not add these additional analyses to the body of text not only as this would lengthen the manuscript considerably and shift its focus on variables that were not part of our research questions, but most importantly because the key results with respect to language group (natives vs. non-native processing) and presence/absence of sound do not change. However, we have incorporated these additional analyses in the R data files, so that interested readers can access this additional information. We attach them as a zipped file to our revised submission.

7.To strengthen the study findings, the authors should at least consider including potential confounding variables whose data was collected in some of the analysis. Recognising this as a limitation is not a good practice, especially when the data is available (i.e., trustworthy is limited).

We thank the reviewer for this comment, which we have addressed in our in-depth response to point 6 above. We have taken on the reviewer's suggestion and repeated all eye-movement analyses by adding all data that we had available (since it was collected, as the reviewer

pointed out), namely the following variables: age, gender, subtitling preferences and education.

8. Even though the paper did a detailed literature review, the introduction or background appear to be too long.

We have now amended the literature review section to better reflect the rationale for carrying out the study, and in doing so we condensed the background information as requested.

Reviewer 3

Accepted with Major revisions. Triangulation for data analysis is compulsory for Mixed methods studies.

Triangulation of the results from different sources (self-reports, eye tracking, and questionnaires) have been conducted through:

- Correlations between English proficiency of Polish participants and their Comprehension and Recall results (Table 3)
- Correlations between various indicators of Cognitive Load, Immersion, and Enjoyment (Table 8)
- Relating results from the qualitative and quantitative methods in the Discussion section

We also added references to the book on research methods by Creswell & Creswell and stressed the mixed methods approach, as suggested by the Reviewer in the manuscript, in the Introduction, Method, Discussion and Conclusion sections. Moreover, we reference Bryman's concept of "completeness" in relation to our mixed methods approach (2006, p.106), in that the interviews are employed to provide richer, more detailed insights into participants' views and their motivations for watching audiovisual content without sound. Below is an example of such additions:

Adopting a mixed-methods approach [15], we collect quantitative data from self-reported immersion, enjoyment and cognitive load questionnaires, comprehension and recall post-tests, as well as eye-tracking data. Additionally, qualitative insights are gathered through semi-structured interviews. Following Creswell & Creswell [15], we use the convergent mixed methods design, combining the results from the quantitative and qualitative data in order to see if the findings from both approaches confirm or disconfirm each other. Semi-structured

interviews are also conducted for the purpose of completeness; i.e., to “bring together a more comprehensive account of the area of enquiry” [62].

Details of questionnaire was not defined properly, was it a self-designed by the research team of the project or adopted? Same observation holds for semi-structured interview. NOTE: If both instruments were adopted then whether reliability and validity of both were tested in the given research context. I don’t find such information in any part of the part of the paper. If not then it must be tested and validated.

The questionnaires consisted of the following: Cognitive Load, Immersion, and Enjoyment.

- Cognitive load was assessed with three relevant indicators from the NASA-TLX instrument (https://humansystems.arc.nasa.gov/groups/TLX/), focused on Difficulty (corresponding to Mental Demand in the NASA-TLX instrument), Effort (corresponding to Effort in the NASA-TLX instrument), and Frustration (corresponding to Frustration Level in the NASA-TLX instrument). Each was measured on a scale of 1 to 7, where “1” represented the lowest value, e.g., lowest effort, and “7” represented the highest value, e.g., highest effort.

The questionnaire using these three Cognitive Load questions was successfully used in previous studies:

Szarkowska, A., & Gerber-Morón, O. (2018). Viewers can keep up with fast subtitles: Evidence from eye movements. PLoS ONE, 13(6). https://doi.org/10.1371/journal.pone.0199331

- Immersion was operationalised as the captivation dimension, as defined and validated by Rigby et al. (2019):

On a scale from 1 to 7, to what extent did the clip hold your attention?
1 means “It did’t hold my attention at all” and 7 means “It held my attention a lot”.

Rigby, J. M., Brumby, D. P., Gould, S. J. J., & Cox, A. L. (2019). Development of a Questionnaire to Measure Immersion in Video Media Proceedings of the 2019 ACM International Conference on Interactive Experiences for TV and Online Video,

- The **Enjoyment** question was created by us and simply asked the participant to state their self-reported level of enjoyment:

On a scale from 1 to 7, how much did you enjoy watching this clip?
1 means “I didn’t enjoy it at all” and 7 means “I enjoyed it very much”.

Following the Reviewer's suggestions, we have added more detailed explanations on the origins of the questions on Cognitive Load and the relevant reference:

Cognitive load was tested using three dimensions derived from from the NASA-TLX instrument [69]: difficulty (equivalent to Mental Demand in the NASA-TLX instrument), effort (corresponding to Effort in the NASA-TLX instrument), and frustration (equivalent to Frustration Level in the NASA-TLX instrument).

To ensure internal consistency, following the Reviewer's suggestion, we ran Cronbach's alpha on the three indicators of Cognitive Load in both conditions. They showed high internal consistency: Frustration, Difficulty, and Effort with sound ON $\alpha = 0.734$, and with sound OFF $\alpha = 0.813$.

We added this paragraph to the manuscript:

To check the internal validity of the three indicators of cognitive load (Difficulty, Effort, Frustration), we conducted the Cronbach's Alpha test. Cronbach Alpha for the sound-on condition was $\alpha = .73$, whereas for the sound-off condition $\alpha = .81$. Overall, the internal validity of our cognitive load measurement instrument was good.

As regards the **interviews**, the questions were designed to be tailored to the particular topic and context of the study. Semi-structured interview questions are not validated because they are tailored to the particular context, topic and participant. Further, to our knowledge no prior studies that investigated the presence/absence of sound have included a post-test interview phase with validated questions and this is the first to explicitly enquire with study participants how reading subtitles on a video without sound affects their viewing experience.

Our goal in this paper was not to create validated instruments for future use, but to assess whether additional comments and insights gained in the interviews would support the quantitative data from questionnaires and eye-tracking analyses and provide a more comprehensive account of participant perceptions. Moreover, we piloted the interview questions on a group of viewers (separate from those of the main study) before the main data collection, and considered the questions effective if they elicited fine-grained responses specifically about the presence/absence of sound. Question structure and order were amended following this piloting phase and the final version is provided in the appendix/supplementary materials.
