# Supplementary material for: Watching subtitled videos with the sound off affects viewers’ comprehension, cognitive load, immersion, enjoyment, and gaze patterns: A mixed-methods eye-tracking study
Source: PLoS One. 2024 Oct 7;19(10):e0306251. doi: 10.1371/journal.pone.0306251 (PMC11458047; doi:10.1371/journal.pone.0306251)
Supplement: S1 Data — (ZIP) [file pone.0306251.s001.zip › Data analysis_Revised after peer review_Feb 2024/2. Tables/Copy of Table 10_Model summaries.docx]

***Table 10.*** *(G)LMMs Results for the eye-tracking analyses on the subtitle region.*

| **Measures** | **Contrasts** | **β** | **SE** | **t** | **p** |
| --- | --- | --- | --- | --- | --- |
| Total  Reading  Time | (Intercept) | 883.20 | 43.31 | 20.390 | **<.0001***** |
|  | Audio (OFF) | 277.48 | 29.48 | 9.413 | **<.0001***** |
|  | Group (AUS) | -22.80 | 44.70 | -0.510 | 0.611 |
|  | Group (PL) | 233.68 | 42.73 | 5.468 | **<.0001***** |
|  | Sound (OFF) × Group (AUS) | 18.02 | 38.38 | 0.469 | 0.639 |
|  | Sound (OFF) × Group (PL) | -30.29 | 36.68 | -0.826 | 0.410 |
| Mean Fixation Duration | (Intercept) | 5.242710 | 0.020119 | 260.591 | **<.0001***** |
|  | Sound (OFF) | 0.044884 | 0.010697 | 4.196 | **<.0001***** |
|  | Group (AUS) | 0.042455 | 0.026614 | 1.595 | 0.113 |
|  | Group (PL) | 0.025916 | 0.025442 | 1.019 | 0.310 |
|  | Sound (OFF) × Group (AUS) | -0.003036 | 0.014198 | -0.214 | 0.831 |
|  | Sound (OFF) × Group (PL) | -0.012810 | 0.013570 | -0.944 | 0.347 |
| Skipping  Rates | (Intercept) | -3.54914 | 0.19925 | -17.813 | **<.0001***** |
|  | Sound (OFF) | -1.19266 | 0.10774 | -11.070 | **<.0001***** |
|  | Group (AUS) | -0.08445 | 0.25217 | -0.335 | 0.73771 |
|  | Group (PL) | -0.90793 | 0.24724 | -3.672 | **0.00024***** |
|  | Sound (OFF) × Group (AUS) | 0.19778 | 0.14396 | 1.374 | 0.16948 |
|  | Sound (OFF) × Group (PL) | 0.19995 | 0.15647 | 1.278 | 0.20129 |
| Crossover Counts | (Intercept) | 1.993605 | 0.035201 | 56.635 | **<.0001***** |
|  | Sound (OFF) | -0.055121 | 0.033129 | -1.664 | 0.09822 |
|  | Group (AUS) | 0.102937 | 0.038603 | 2.667 | **0.00854**** |
|  | Group (PL) | -0.055338 | 0.036899 | -1.500 | 0.13587 |
|  | Sound (OFF) × Group (AUS) | 0.008851 | 0.044669 | 0.198 | 0.84321 |
|  | Sound (OFF) × Group (PL) | -0.081598 | 0.042733 | -1.910 | 0.05810 |
