# Supplementary material for: Watching subtitled videos with the sound off affects viewers’ comprehension, cognitive load, immersion, enjoyment, and gaze patterns: A mixed-methods eye-tracking study
Source: PLoS One. 2024 Oct 7;19(10):e0306251. doi: 10.1371/journal.pone.0306251 (PMC11458047; doi:10.1371/journal.pone.0306251)
Supplement: S1 Data — (ZIP) [file pone.0306251.s001.zip › Data analysis_Revised after peer review_Feb 2024/2. Tables/Copy of Table 9_means and SD_eyetracking measures.docx]

**Table 9.**

*Means and standard deviations of eye-tracking measures on the subtitle area by Audio condition (ON/OFF) and Group (AUS, UK, PL).*

|  | **Mean  Fixation Durations (ms)** | | | **Total**  **Reading**  **Times**  **(ms)** | | | | **Skipping**  **Rates** | | | | **Crossover  Counts** | | | |
| --- | --- | --- | --- | --- | --- | --- | --- | --- | --- | --- | --- | --- | --- | --- | --- |
| **Sound** | AUS (SD) | UK (SD) | PL (SD) | | AUS (SD) | UK  (SD) | PL (SD) | | AUS (SD) | UK  (SD) | PL (SD) | | AUS (SD) | UK (SD) | PL (SD) |
| **ON** | 207 (65) | 197 (60) | 201 (55) | | 888 (623) | 922 (633) | 1132 (724) | | 0.063 (0.243) | 0.074 (0.261) | 0.036 (0.187) | | 2.122 (0.919) | 2.018 (0.830) | 1.960 (0.875) |
| **OFF** | 216 (70) | 205 (62) | 207 (53) | | 1167 (777) | 1165 (731) | 1379 (796) | | 0.029 (0.167) | 0.028 (0.165) | 0.016 (0.124) | | 2.088 (1.032) | 1.976 (0.934) | 1.840 (0.913) |
