# Supplementary material for: Watching subtitled videos with the sound off affects viewers’ comprehension, cognitive load, immersion, enjoyment, and gaze patterns: A mixed-methods eye-tracking study
Source: PLoS One. 2024 Oct 7;19(10):e0306251. doi: 10.1371/journal.pone.0306251 (PMC11458047; doi:10.1371/journal.pone.0306251)
Supplement: S1 Data — (ZIP) [file pone.0306251.s001.zip › Data analysis_Revised after peer review_Feb 2024/CC_revised/CC_global_Saccade_Exp2.html]

Crossover Counts\_Subtitle-level


# Crossover Counts\_Subtitle-level

#### VR

#### Feb 2024

```
sessionInfo() # R version 4.3.2 (2023-10-31 ucrt)

# load libraries
library(lattice)
library(lme4)
library(lmerTest)
library(emmeans)
library(sjPlot)
library(MASS)
library(tidyverse)
library(ggplot2)
library(pastecs)
library(udpipe)
library(optimx)
library(psych)
library(nlme)
```

```
global <- read.csv ("Exp2_Global_SaccadeReport_merged.csv", header=T) # 462,123 obs, 59 vars
```

**NOTES ABOUT DATA EXCLUSIONS**:

- PL42, PL47, UK19, UK44, AUS05, AUS07, AUS51 are excluded due to
  low eye-tracking data quality.
- UK10, UK18, AUS12, AUS49, AUS51, AUS56 are excluded because they
  are not native speakers of English, or because they are
  *American* English native speakers (not British or
  Australian).

## Global dataset processing

```
glimpse(global) 
names(global)

global$subj  = as.factor(global$subj)
global$audio.cond  = as.factor(global$audio.cond)
global$native.lang = as.factor(global$native.lang)
global$group  = as.factor(global$group)
global$unique.sub.id = as.factor(global$unique.sub.id)
global$sub.cond = as.factor(global$sub.cond)
global$sub.id  = as.factor(global$sub.id)

global$CURRENT_SAC_DIRECTION = as.factor(global$CURRENT_SAC_DIRECTION)
global$sacc_start_IA_label  = as.factor(global$sacc_start_IA_label)
global$sacc_end_IA_label  = as.factor(global$sacc_end_IA_label)
```

## Data Exclusions & variable creation

```
cc.df <- data.frame(global) 

cc.df = cc.df [!cc.df$subj %in% c("PL42", "PL47", "UK19", "UK44", "AUS05", "AUS07","AUS51"),] # 445,123 rows
cc.df = cc.df [!cc.df$subj %in% c("UK10", "UK18", "AUS12", "AUS49", "AUS51", "AUS56"),] # 430,872 rows
cc.df<-cc.df[cc.df$sub.cond=="congruent",] # 292,480 rows

summary(cc.df$CURRENT_SAC_AMPLITUDE)
summary(cc.df$CURRENT_SAC_DIRECTION) 
cc.df<-subset(cc.df, CURRENT_SAC_DIRECTION != ".") # 285,214 rows
cc.df<-subset(cc.df, sacc_start_IA_label != ".") # 280,175 rows
cc.df<-subset(cc.df, sacc_end_IA_label != ".") # 276,238 rows

cc.df<-subset(cc.df, (sacc_start_IA_label == "Sub_IA" & sacc_end_IA_label == "Video_IA")|
                   (sacc_start_IA_label == "Video_IA" & sacc_end_IA_label == "Sub_IA"))  # 66,714 rows

# create  variable 'crossover counts' by aggregating saccade amplitude
glimpse(cc.df)
cross.df<-aggregate(CURRENT_SAC_AMPLITUDE~subj+group+native.lang+unique.sub.id+audio.cond+TRIAL_INDEX+age+education+gender+AVT_Pref_bin,
                   data=cc.df,length) # 33,439 rows

glimpse(cross.df) # 33,439 obs, 11 vars
names(cross.df)
names(cross.df)[11] <- "cross.count"
cross.df$cross.count = as.numeric(cross.df$cross.count)
```

## Crossover Counts (CC) Descriptives & Visualisations

```
# mean and sd
cat("\n mean", mean(cross.df$cross.count, na.rm=T)) #   mean 1.995096
cat("\n sd ", sd(cross.df$cross.count, na.rm=T)) #  sd  0.9236865
```

```
##    Min. 1st Qu.  Median    Mean 3rd Qu.    Max. 
##   1.000   1.000   2.000   1.995   2.000  11.000
```

```
##    vars     n mean   sd median trimmed mad min max range skew kurtosis   se
## X1    1 33439    2 0.92      2    1.88   0   1  11    10 1.39     3.56 0.01
```

```
## # A tibble: 2 × 2
##   audio.cond mean_CrossNum
##   <fct>              <dbl>
## 1 OFF                 1.96
## 2 ON                  2.03
```

```
## # A tibble: 3 × 2
##   group avg_CrossNum
##   <fct>        <dbl>
## 1 AUS           2.11
## 2 PL            1.90
## 3 UK            2.00
```

| Audio Condition | Language Group | Mean | SD |
| --- | --- | --- | --- |
| OFF | AUS | 2.088 | 1.032 |
| OFF | PL | 1.840 | 0.913 |
| OFF | UK | 1.976 | 0.934 |
| ON | AUS | 2.122 | 0.919 |
| ON | PL | 1.960 | 0.875 |
| ON | UK | 2.018 | 0.830 |

## Contrast Setting & releveling

```
# check contrasts
contrasts(cross.df$audio.cond) # dummy/treatment coding

# sound ON as the reference level
cross.df$audio.cond<-relevel(cross.df$audio.cond,"ON")

# UK as the reference level
cross.df$group<-relevel(cross.df$group,"UK")
```

# Modelling for CC (global analysis, CC>0)

Intermediary steps, including those run with uncorrelated slopes, are
omitted for brevity.

```
# failed to converge or singular fits, unless otherwise marked
cc.lmer.01 = lmer(cross.count ~ audio.cond*group + (1+audio.cond*group|subj) + (1+audio.cond*group|unique.sub.id), data=cross.df) 

cc.lmer.02 = lmer(cross.count ~ audio.cond*group + (1+audio.cond*group|subj) + (1+audio.cond+group|unique.sub.id), data=cross.df) 

cc.lmer.03 = lmer(cross.count ~ audio.cond*group + (1+audio.cond*group|subj)+(1+audio.cond|unique.sub.id), data=cross.df) 

cc.lmer.04 = lmer(cross.count ~ audio.cond*group + (1+audio.cond*group|subj) + (1|unique.sub.id), data=cross.df) 

cc.lmer.05 = lmer(cross.count ~ audio.cond*group + (1+audio.cond|subj)+(1+audio.cond*group|unique.sub.id), data=cross.df) 

cc.lmer.06 = lmer(cross.count ~ audio.cond*group + (1|subj)+(1+audio.cond*group|unique.sub.id), data=cross.df) 

cc.lmer.07 = lmer(cross.count ~ audio.cond*group + (1+audio.cond+group|subj)+(1+audio.cond+group|unique.sub.id), data=cross.df) 

cc.lmer.08 = lmer(cross.count ~ audio.cond*group + (1+audio.cond+group|subj)+(1+audio.cond|unique.sub.id), data=cross.df) 

cc.lmer.09 = lmer(cross.count ~ audio.cond*group + (1+audio.cond+group|subj) + (1|unique.sub.id), data=cross.df)

cc.lmer.10 = lmer(cross.count ~ audio.cond*group + (1+audio.cond|subj)+(1+audio.cond+group|unique.sub.id), data=cross.df) 

cc.lmer.11 = lmer(cross.count ~ audio.cond*group + (1+group|subj)+(1+audio.cond+group|unique.sub.id), data=cross.df)

cc.lmer.12 = lmer(cross.count ~ audio.cond*group + (1|subj)+(1+audio.cond+group|unique.sub.id), data=cross.df) 

cc.lmer.13 = lmer(cross.count ~ audio.cond*group + (1+audio.cond|subj)+ (1+audio.cond|unique.sub.id), data=cross.df) 

cc.lmer.14 = lmer(cross.count ~ audio.cond*group + (1+group|subj)+ (1+group|unique.sub.id), data=cross.df) 

cc.lmer.15 = lmer(cross.count ~ audio.cond*group + (1+audio.cond|subj)+(1+group|unique.sub.id) , data=cross.df)

cc.lmer.16 = lmer(cross.count ~ audio.cond*group + (1+group|subj)+(1+audio.cond|unique.sub.id) , data=cross.df)

cc.lmer.17 = lmer(cross.count ~ audio.cond*group + (1+audio.cond|subj) + (1|unique.sub.id), data=cross.df) # fitted OK

cc.lmer.18= lmer(cross.count ~ audio.cond*group + (1+group|subj) + (1|unique.sub.id), data=cross.df)

cc.lmer.19 = lmer(cross.count ~ audio.cond*group + (1|subj)+(1+audio.cond|unique.sub.id), data=cross.df) # fitted OK

cc.lmer.20 = lmer(cross.count ~ audio.cond*group + (1|subj)+(1+group|unique.sub.id), data=cross.df) 

cc.lmer.min = lmer(cross.count ~ audio.cond*group + (1|subj) + (1|unique.sub.id), data=cross.df) # fitted OK
```

```
anova(cc.lmer.min, cc.lmer.17, cc.lmer.19) # cc.lmer.17
```

```
## Data: cross.df
## Models:
## cc.lmer.min: cross.count ~ audio.cond * group + (1 | subj) + (1 | unique.sub.id)
## cc.lmer.17: cross.count ~ audio.cond * group + (1 + audio.cond | subj) + (1 | unique.sub.id)
## cc.lmer.19: cross.count ~ audio.cond * group + (1 | subj) + (1 + audio.cond | unique.sub.id)
##             npar   AIC   BIC logLik deviance  Chisq Df Pr(>Chisq)    
## cc.lmer.min    9 82438 82514 -41210    82420                         
## cc.lmer.17    11 82225 82318 -41102    82203 216.79  2  < 2.2e-16 ***
## cc.lmer.19    11 82288 82381 -41133    82266   0.00  0               
## ---
## Signif. codes:  0 '***' 0.001 '**' 0.01 '*' 0.05 '.' 0.1 ' ' 1
```

# Final CC Model

```
#  run PCA
summary(rePCA(cc.lmer.17))
```

```
## $unique.sub.id
## Importance of components:
##                          [,1]
## Standard deviation     0.4844
## Proportion of Variance 1.0000
## Cumulative Proportion  1.0000
## 
## $subj
## Importance of components:
##                          [,1]   [,2]
## Standard deviation     0.2341 0.2003
## Proportion of Variance 0.5774 0.4226
## Cumulative Proportion  0.5774 1.0000
```

```
# model details
summary(cc.lmer.17)
```

```
## Linear mixed model fit by REML. t-tests use Satterthwaite's method [
## lmerModLmerTest]
## Formula: cross.count ~ audio.cond * group + (1 + audio.cond | subj) +  
##     (1 | unique.sub.id)
##    Data: cross.df
## 
## REML criterion at convergence: 82234.4
## 
## Scaled residuals: 
##     Min      1Q  Median      3Q     Max 
## -3.9637 -0.6891 -0.0185  0.4500  9.5807 
## 
## Random effects:
##  Groups        Name          Variance Std.Dev. Corr 
##  unique.sub.id (Intercept)   0.15261  0.3907        
##  subj          (Intercept)   0.02825  0.1681        
##                audio.condOFF 0.03350  0.1830   -0.13
##  Residual                    0.65038  0.8065        
## Number of obs: 33439, groups:  unique.sub.id, 364; subj, 156
## 
## Fixed effects:
##                          Estimate Std. Error         df t value Pr(>|t|)    
## (Intercept)              1.993605   0.035201 298.637530  56.635  < 2e-16 ***
## audio.condOFF           -0.055121   0.033129 150.609557  -1.664  0.09822 .  
## groupAUS                 0.102937   0.038603 144.773776   2.667  0.00854 ** 
## groupPL                 -0.055338   0.036899 144.787814  -1.500  0.13587    
## audio.condOFF:groupAUS   0.008851   0.044669 149.625499   0.198  0.84321    
## audio.condOFF:groupPL   -0.081598   0.042733 150.227916  -1.910  0.05810 .  
## ---
## Signif. codes:  0 '***' 0.001 '**' 0.01 '*' 0.05 '.' 0.1 ' ' 1
## 
## Correlation of Fixed Effects:
##             (Intr) ad.OFF grpAUS gropPL a.OFF:A
## audi.cndOFF -0.207                             
## groupAUS    -0.603  0.189                      
## groupPL     -0.631  0.198  0.575               
## ad.cOFF:AUS  0.154 -0.741 -0.253 -0.147        
## ad.cnOFF:PL  0.161 -0.775 -0.147 -0.253  0.575
```

```
# get table of model coefficients 
tab_model(cc.lmer.17, file = "CC_lme_cc.lmer.17.doc")
```

|  | cross.count | | |
| --- | --- | --- | --- |
| Predictors | Estimates | CI | p |
| (Intercept) | 1.99 | 1.92 – 2.06 | **<0.001** |
| audio cond [OFF] | -0.06 | -0.12 – 0.01 | 0.096 |
| group [AUS] | 0.10 | 0.03 – 0.18 | **0.008** |
| group [PL] | -0.06 | -0.13 – 0.02 | 0.134 |
| audio cond [OFF] × group [AUS] | 0.01 | -0.08 – 0.10 | 0.843 |
| audio cond [OFF] × group [PL] | -0.08 | -0.17 – 0.00 | 0.056 |
| Random Effects | | | |
| σ^2^ | 0.65 | | |
| τ_00_ _unique.sub.id_ | 0.15 | | || τ_00_ _subj_ | 0.03 | | || τ_11_ _subj.audio.condOFF_ | 0.03 | | || ρ_01_ _subj_ | -0.13 | | || ICC | 0.23 | | || N _subj_ | 156 | | || N _unique.sub.id_ | 364 | | || Observations | 33439 | | |
| Marginal R^2^ / Conditional R^2^ | 0.011 / 0.238 | | |

## Extract pairwise comparisons of means

```
# Obtain marginal means of the predictions
library(emmeans)
emm <- emmeans(cc.lmer.17, ~ audio.cond | group)
```

```
# plot  marginal means
plot(emm)
```

```
emmip(emm, group ~ audio.cond, CIs = TRUE)
```

```
emm1 = emmeans(cc.lmer.17, specs = pairwise ~ audio.cond:group)
emm1
```

```
## $emmeans
##  audio.cond group emmean     SE  df asymp.LCL asymp.UCL
##  ON         UK      1.99 0.0352 Inf      1.92      2.06
##  OFF        UK      1.94 0.0430 Inf      1.85      2.02
##  ON         AUS     2.10 0.0330 Inf      2.03      2.16
##  OFF        AUS     2.05 0.0400 Inf      1.97      2.13
##  ON         PL      1.94 0.0310 Inf      1.88      2.00
##  OFF        PL      1.80 0.0371 Inf      1.73      1.87
## 
## Degrees-of-freedom method: asymptotic 
## Confidence level used: 0.95 
## 
## $contrasts
##  contrast          estimate     SE  df z.ratio p.value
##  ON UK - OFF UK    0.055121 0.0331 Inf   1.664  0.5559
##  ON UK - ON AUS   -0.102937 0.0386 Inf  -2.667  0.0820
##  ON UK - OFF AUS  -0.056667 0.0447 Inf  -1.267  0.8028
##  ON UK - ON PL     0.055338 0.0369 Inf   1.500  0.6645
##  ON UK - OFF PL    0.192057 0.0421 Inf   4.557  0.0001
##  OFF UK - ON AUS  -0.158058 0.0459 Inf  -3.446  0.0075
##  OFF UK - OFF AUS -0.111788 0.0511 Inf  -2.187  0.2437
##  OFF UK - ON PL    0.000217 0.0444 Inf   0.005  1.0000
##  OFF UK - OFF PL   0.136936 0.0489 Inf   2.801  0.0573
##  ON AUS - OFF AUS  0.046271 0.0300 Inf   1.544  0.6358
##  ON AUS - ON PL    0.158276 0.0348 Inf   4.545  0.0001
##  ON AUS - OFF PL   0.294995 0.0404 Inf   7.311  <.0001
##  OFF AUS - ON PL   0.112005 0.0415 Inf   2.700  0.0752
##  OFF AUS - OFF PL  0.248724 0.0462 Inf   5.382  <.0001
##  ON PL - OFF PL    0.136719 0.0270 Inf   5.064  <.0001
## 
## Degrees-of-freedom method: asymptotic 
## P value adjustment: tukey method for comparing a family of 6 estimates
```

```
emm1$contrasts %>%
     summary(infer = TRUE)
```

```
##  contrast          estimate     SE  df asymp.LCL asymp.UCL z.ratio p.value
##  ON UK - OFF UK    0.055121 0.0331 Inf  -0.03929   0.14953   1.664  0.5559
##  ON UK - ON AUS   -0.102937 0.0386 Inf  -0.21295   0.00707  -2.667  0.0820
##  ON UK - OFF AUS  -0.056667 0.0447 Inf  -0.18407   0.07074  -1.267  0.8028
##  ON UK - ON PL     0.055338 0.0369 Inf  -0.04981   0.16049   1.500  0.6645
##  ON UK - OFF PL    0.192057 0.0421 Inf   0.07194   0.31217   4.557  0.0001
##  OFF UK - ON AUS  -0.158058 0.0459 Inf  -0.28878  -0.02733  -3.446  0.0075
##  OFF UK - OFF AUS -0.111788 0.0511 Inf  -0.25744   0.03387  -2.187  0.2437
##  OFF UK - ON PL    0.000217 0.0444 Inf  -0.12644   0.12687   0.005  1.0000
##  OFF UK - OFF PL   0.136936 0.0489 Inf  -0.00238   0.27625   2.801  0.0573
##  ON AUS - OFF AUS  0.046271 0.0300 Inf  -0.03914   0.13168   1.544  0.6358
##  ON AUS - ON PL    0.158276 0.0348 Inf   0.05903   0.25752   4.545  0.0001
##  ON AUS - OFF PL   0.294995 0.0404 Inf   0.18000   0.40998   7.311  <.0001
##  OFF AUS - ON PL   0.112005 0.0415 Inf  -0.00621   0.23022   2.700  0.0752
##  OFF AUS - OFF PL  0.248724 0.0462 Inf   0.11702   0.38042   5.382  <.0001
##  ON PL - OFF PL    0.136719 0.0270 Inf   0.05978   0.21366   5.064  <.0001
## 
## Degrees-of-freedom method: asymptotic 
## Confidence level used: 0.95 
## Conf-level adjustment: tukey method for comparing a family of 6 estimates 
## P value adjustment: tukey method for comparing a family of 6 estimates
```

## Covariates addition

```
glimpse(cross.df) 
cross.df$gender  = as.factor(cross.df$gender)
cross.df$AVT_Pref_bin  = as.factor(cross.df$AVT_Pref_bin)

levels(cross.df$gender)
levels(cross.df$AVT_Pref_bin)
```

```
# fit model
cc.lmer.17.extra = lmer(cross.count ~ audio.cond*group + gender + age + education + AVT_Pref_bin + (1+audio.cond|subj) + (1|unique.sub.id), data=cross.df) 
  
summary(cc.lmer.17.extra) # t-values are small, none of the covariates are significant
```

```
## Linear mixed model fit by REML. t-tests use Satterthwaite's method [
## lmerModLmerTest]
## Formula: cross.count ~ audio.cond * group + gender + age + education +  
##     AVT_Pref_bin + (1 + audio.cond | subj) + (1 | unique.sub.id)
##    Data: cross.df
## 
## REML criterion at convergence: 82260.2
## 
## Scaled residuals: 
##     Min      1Q  Median      3Q     Max 
## -3.9680 -0.6894 -0.0196  0.4507  9.5815 
## 
## Random effects:
##  Groups        Name          Variance Std.Dev. Corr 
##  unique.sub.id (Intercept)   0.15254  0.3906        
##  subj          (Intercept)   0.02800  0.1673        
##                audio.condOFF 0.03343  0.1828   -0.12
##  Residual                    0.65038  0.8065        
## Number of obs: 33439, groups:  unique.sub.id, 364; subj, 156
## 
## Fixed effects:
##                          Estimate Std. Error         df t value Pr(>|t|)    
## (Intercept)              2.168737   0.105943 153.360897  20.471   <2e-16 ***
## audio.condOFF           -0.055075   0.033103 150.653986  -1.664   0.0982 .  
## groupAUS                 0.081321   0.042266 143.391012   1.924   0.0563 .  
## groupPL                 -0.045402   0.037896 140.802478  -1.198   0.2329    
## genderM                  0.029369   0.035202 141.919294   0.834   0.4055    
## genderNB                 0.054705   0.084586 139.528042   0.647   0.5189    
## age                     -0.003012   0.002515 141.442274  -1.197   0.2331    
## education               -0.003450   0.006295 141.168246  -0.548   0.5845    
## AVT_Pref_binSubs        -0.056164   0.045384 140.246914  -1.238   0.2180    
## audio.condOFF:groupAUS   0.008760   0.044634 149.664520   0.196   0.8447    
## audio.condOFF:groupPL   -0.081628   0.042699 150.269731  -1.912   0.0578 .  
## ---
## Signif. codes:  0 '***' 0.001 '**' 0.01 '*' 0.05 '.' 0.1 ' ' 1
## 
## Correlation of Fixed Effects:
##             (Intr) ad.OFF grpAUS gropPL gendrM gndrNB age    eductn AVT_P_
## audi.cndOFF -0.065                                                        
## groupAUS    -0.463  0.167                                                 
## groupPL     -0.228  0.185  0.508                                          
## genderM      0.032  0.000  0.010  0.001                                   
## genderNB    -0.123  0.005  0.236  0.152  0.073                            
## age         -0.062  0.003  0.222 -0.152 -0.281  0.136                     
## education   -0.656 -0.002  0.091  0.095  0.074 -0.071 -0.582              
## AVT_Prf_bnS -0.505 -0.002  0.081  0.039 -0.053  0.060  0.054  0.086       
## ad.cOFF:AUS  0.049 -0.741 -0.223 -0.137  0.000 -0.004 -0.002  0.002  0.001
## ad.cnOFF:PL  0.052 -0.775 -0.130 -0.236  0.000 -0.004 -0.001 -0.001  0.002
##             a.OFF:A
## audi.cndOFF        
## groupAUS           
## groupPL            
## genderM            
## genderNB           
## age                
## education          
## AVT_Prf_bnS        
## ad.cOFF:AUS        
## ad.cnOFF:PL  0.575
```

```
# model explains virtually no additional variance at all
tab_model(cc.lmer.17.extra,file = "CC_lme_cc.lmer.17.extra.doc")
```

|  | cross.count | | |
| --- | --- | --- | --- |
| Predictors | Estimates | CI | p |
| (Intercept) | 2.17 | 1.96 – 2.38 | **<0.001** |
| audio cond [OFF] | -0.06 | -0.12 – 0.01 | 0.096 |
| group [AUS] | 0.08 | -0.00 – 0.16 | 0.054 |
| group [PL] | -0.05 | -0.12 – 0.03 | 0.231 |
| gender [M] | 0.03 | -0.04 – 0.10 | 0.404 |
| gender [NB] | 0.05 | -0.11 – 0.22 | 0.518 |
| age | -0.00 | -0.01 – 0.00 | 0.231 |
| education | -0.00 | -0.02 – 0.01 | 0.584 |
| AVT Pref bin [Subs] | -0.06 | -0.15 – 0.03 | 0.216 |
| audio cond [OFF] × group [AUS] | 0.01 | -0.08 – 0.10 | 0.844 |
| audio cond [OFF] × group [PL] | -0.08 | -0.17 – 0.00 | 0.056 |
| Random Effects | | | |
| σ^2^ | 0.65 | | |
| τ_00_ _unique.sub.id_ | 0.15 | | || τ_00_ _subj_ | 0.03 | | || τ_11_ _subj.audio.condOFF_ | 0.03 | | || ρ_01_ _subj_ | -0.12 | | || ICC | 0.23 | | || N _subj_ | 156 | | || N _unique.sub.id_ | 364 | | || Observations | 33439 | | |
| Marginal R^2^ / Conditional R^2^ | 0.013 / 0.239 | | |

```
# compare models
anova(cc.lmer.17,cc.lmer.17.extra) # original model (mod 17) has lower AIC & BIC
```

```
## refitting model(s) with ML (instead of REML)
```

```
## Data: cross.df
## Models:
## cc.lmer.17: cross.count ~ audio.cond * group + (1 + audio.cond | subj) + (1 | unique.sub.id)
## cc.lmer.17.extra: cross.count ~ audio.cond * group + gender + age + education + AVT_Pref_bin + (1 + audio.cond | subj) + (1 | unique.sub.id)
##                  npar   AIC   BIC logLik deviance  Chisq Df Pr(>Chisq)
## cc.lmer.17         11 82225 82318 -41102    82203                     
## cc.lmer.17.extra   16 82230 82364 -41099    82198 5.6997  5     0.3365
```
