# Supplementary material for: Watching subtitled videos with the sound off affects viewers’ comprehension, cognitive load, immersion, enjoyment, and gaze patterns: A mixed-methods eye-tracking study
Source: PLoS One. 2024 Oct 7;19(10):e0306251. doi: 10.1371/journal.pone.0306251 (PMC11458047; doi:10.1371/journal.pone.0306251)
Supplement: S1 Data — (ZIP) [file pone.0306251.s001.zip › Data analysis_Revised after peer review_Feb 2024/Chronbach's alpha/CL_Chronbach.html]

Chronbach’s alpha


# Chronbach’s alpha

#### VR

#### Feb 2024

```
sessionInfo() # R version  4.3.2 (2023-10-31 ucrt)

# load libraries
library(tidyverse)
library(ltm)
```

```
# import df
alpha.df <- read.csv ("Questionnaire_dataset_Chronbach.csv", header=T) # 161 obs, 11 vars

# inspect df
glimpse(alpha.df)
names(alpha.df)
```

```
# subset df as to have only cognitive load (CL) indicators (sound ON condition)
CL.ON.df <- subset(alpha.df, select=c(6,8,10))

# subset df as to have only cognitive load (CL) indicators (sound OFF condition)
CL.OFF.df <- subset(alpha.df, select=c(7,9,11))
```

```
# calculate cronbach's alpha for Cognitive Load sound ON
cronbach.alpha(CL.ON.df, CI=TRUE, standardized=TRUE) # alpha: 0.749
```

```
## 
## Standardized Cronbach's alpha for the 'CL.ON.df' data-set
## 
## Items: 3
## Sample units: 161
## alpha: 0.749
## 
## Bootstrap 95% CI based on 1000 samples
##  2.5% 97.5% 
## 0.659 0.818
```

```
# calculate cronbach's alpha for Cognitive Load sound OFF
cronbach.alpha(CL.OFF.df, CI=TRUE, standardized=TRUE) # alpha: 0.813
```

```
## 
## Standardized Cronbach's alpha for the 'CL.OFF.df' data-set
## 
## Items: 3
## Sample units: 161
## alpha: 0.813
## 
## Bootstrap 95% CI based on 1000 samples
##  2.5% 97.5% 
## 0.754 0.858
```

```
# subset df as to have only ENJOYMENT and CAPTIVATION (sound ON condition)
names(alpha.df)
EnjCap.ON.df <- subset(alpha.df, select=c(2,4))

# subset df as to have only ENJOYMENT and CAPTIVATION (sound OFF condition)
names(alpha.df)
EnjCap.OFF.df <- subset(alpha.df, select=c(3,5))
```

```
# calculate cronbach's alphaENJOYMENT and CAPTIVATION (sound ON condition)
cronbach.alpha(EnjCap.ON.df, CI=TRUE, standardized=TRUE) # alpha: 0.924
```

```
## 
## Standardized Cronbach's alpha for the 'EnjCap.ON.df' data-set
## 
## Items: 2
## Sample units: 161
## alpha: 0.924
## 
## Bootstrap 95% CI based on 1000 samples
##  2.5% 97.5% 
## 0.885 0.950
```

```
# calculate cronbach's alpha ENJOYMENT and CAPTIVATION (sound OFF condition)
cronbach.alpha(EnjCap.OFF.df, CI=TRUE, standardized=TRUE) # alpha: 0.85
```

```
## 
## Standardized Cronbach's alpha for the 'EnjCap.OFF.df' data-set
## 
## Items: 2
## Sample units: 161
## alpha: 0.85
## 
## Bootstrap 95% CI based on 1000 samples
##  2.5% 97.5% 
## 0.787 0.897
```
