# Supplementary material for: Watching subtitled videos with the sound off affects viewers’ comprehension, cognitive load, immersion, enjoyment, and gaze patterns: A mixed-methods eye-tracking study
Source: PLoS One. 2024 Oct 7;19(10):e0306251. doi: 10.1371/journal.pone.0306251 (PMC11458047; doi:10.1371/journal.pone.0306251)
Supplement: S1 Data — (ZIP) [file pone.0306251.s001.zip › Data analysis_Revised after peer review_Feb 2024/MFD_revised/MFD_global_IA_Exp2.html]

MFD\_Subtitle-level


# MFD\_Subtitle-level

#### VR

#### Feb 2024

```
sessionInfo() # R version  4.3.2 (2023-10-31 ucrt)

# load libraries
library(lattice)
library(lme4)
library(lmerTest)
library(emmeans)
library(sjPlot)
library(MASS)
library(tidyverse)
library(ggplot2)
library(pastecs)
library(udpipe)
library(optimx)
library(psych)
library(nlme)
```

```
# import df (global IA report)
global <- read.csv ("Exp2_Global_IA report_merged.csv", header=T) # 123,032 obs, 75 vars
```

**NOTES ABOUT DATA EXCLUSIONS**:

- PL42, PL47, UK19, UK44, AUS05, AUS07, AUS51 are excluded due to
  low eye-tracking data quality.
- UK10, UK18, AUS12, AUS49, AUS51, AUS56 are excluded because they
  are not native speakers of English, or because they are
  *American* English native speakers (not British or
  Australian).

## Global dataset processing

```
summary(global)
glimpse(global)
names(global)

global$subj  = as.factor(global$subj)
global$unique.sub.id = as.factor(global$unique.sub.id)
global$audio.cond  = as.factor(global$audio.cond)
global$group  = as.factor(global$group)
global$sub.cond = as.factor(global$sub.cond)
global$sub.id  = as.factor(global$sub.id)
global$IA_LABEL = as.factor(global$IA_LABEL)
```

## Data exclusions & variable creation

```
# create variable
global$MFD <- global$IA_DWELL_TIME/global$IA_FIXATION_COUNT # 76 vars
global <- subset(global, select=c(1:18, 76, 19:75))
summary(global$MFD)

mfd.df <- data.frame(global)  # 123,032 rows

mfd.df = mfd.df [!mfd.df$subj %in% c("PL42", "PL47", "UK19", "UK44", "AUS05", "AUS07","AUS51"),] # 117,936 rows
mfd.df = mfd.df [!mfd.df$subj %in% c("UK10", "UK18", "AUS12", "AUS49", "AUS51", "AUS56"),] # 114,296 rows
mfd.df<-mfd.df[mfd.df$sub.cond=="congruent",] # 76,172 rows

summary(mfd.df$MFD) # 5822 NAs
mfd.df = mfd.df[mfd.df$MFD!="NaN",] # 70,350 rows
summary(mfd.df$MFD)
```

# IA = SUB Analysis

MFD analysis run on the subtitle region.

```
mfd.df_sub<-mfd.df[mfd.df$IA_LABEL=="Sub_IA",] # 36,591 obs, 76 vars
```

## MFD Descriptives & Visualisations

```
cat("\n mean", mean(mfd.df_sub$MFD, na.rm=T)) #  mean 205.4087
cat("\n sd", sd(mfd.df_sub$MFD, na.rm=T)) #  sd 60.89893
```

```
##    vars     n   mean   sd median trimmed  mad min max range skew kurtosis   se
## X1    1 36591 205.41 60.9 194.89  198.77 45.8  61 796   735 2.08     9.56 0.32
```

```
##    Min. 1st Qu.  Median    Mean 3rd Qu.    Max. 
##    61.0   180.8   226.0   256.5   303.8   799.0
```

```
## # A tibble: 2 × 2
##   audio.cond avg_MFD
##   <fct>        <dbl>
## 1 OFF           209.
## 2 ON            202.
```

```
## # A tibble: 3 × 2
##   group avg_MFD
##   <fct>   <dbl>
## 1 AUS      211.
## 2 PL       204.
## 3 UK       201.
```

| Audio Condition | Language Group | Mean | SD |
| --- | --- | --- | --- |
| OFF | AUS | 215.83 | 69.56 |
| OFF | PL | 206.58 | 53.25 |
| OFF | UK | 204.89 | 62.37 |
| ON | AUS | 206.64 | 65.33 |
| ON | PL | 200.58 | 54.63 |
| ON | UK | 196.54 | 60.13 |

## Contrast Setting & releveling

```
# check contrasts
contrasts(mfd.df_sub$audio.cond) # dummy/treatment coding

# sound ON as the reference level
mfd.df_sub$audio.cond<-relevel(mfd.df_sub$audio.cond,"ON")  

# UK as the reference level (language variety group)
mfd.df_sub$group<-relevel(mfd.df_sub$group,"UK")
```

# Modelling for MFD (global analysis, IA = SUB, MFD>0)

Intermediary steps, including those run with uncorrelated slopes, are
omitted for brevity.

```
s.lmer.max = lmer(log(MFD) ~ audio.cond*group + (1+audio.cond*group|subj) + (1+audio.cond*group|unique.sub.id), data=mfd.df_sub) 

s.lmer.min = lmer(log(MFD) ~ audio.cond*group + (1|subj) + (1|unique.sub.id), data=mfd.df_sub) 
summary(s.lmer.min)
```

```
# failed to converge or singular fits, unless marked otherwise
s.lmer.01 = lmer(log(MFD) ~ audio.cond*group +(1+audio.cond*group|subj)+ (1+audio.cond|unique.sub.id), data=mfd.df_sub) 

s.lmer.02 = lmer(log(MFD) ~ audio.cond*group +(1+audio.cond*group|subj)+ (1+group|unique.sub.id), data=mfd.df_sub) 

s.lmer.03 = lmer(log(MFD) ~ audio.cond*group + (1+audio.cond*group|subj) + (1|unique.sub.id), data=mfd.df_sub) 


s.lmer.04 = lmer(log(MFD) ~ audio.cond*group +(1+audio.cond+group|subj)+ (1+audio.cond+group|unique.sub.id), data=mfd.df_sub) 

s.lmer.05 = lmer(log(MFD) ~ audio.cond*group +(1+audio.cond+group|subj)+ (1+audio.cond|unique.sub.id), data=mfd.df_sub) 

s.lmer.06 = lmer(log(MFD) ~ audio.cond*group +(1+audio.cond+group|subj)+ (1+group|unique.sub.id), data=mfd.df_sub) 

s.lmer.07 = lmer(log(MFD) ~ audio.cond*group +(1+audio.cond+group|subj)+ (1|unique.sub.id), data=mfd.df_sub) 

s.lmer.08 = lmer(log(MFD) ~ audio.cond*group + (1+audio.cond|subj) + (1|unique.sub.id), data=mfd.df_sub) # fitted OK

s.lmer.09 = lmer(log(MFD) ~ audio.cond*group + (1+group|subj) + (1|unique.sub.id), data=mfd.df_sub)


s.lmer.10 = lmer(log(MFD) ~ audio.cond*group+(1+audio.cond|subj)+(1+audio.cond*group|unique.sub.id), data=mfd.df_sub) 

s.lmer.11 = lmer(log(MFD) ~ audio.cond*group+(1+audio.cond|subj)+(1+audio.cond+group|unique.sub.id), data=mfd.df_sub)

s.lmer.12 = lmer(log(MFD) ~ audio.cond*group+(1+audio.cond|subj)+(1+audio.cond|unique.sub.id), data=mfd.df_sub) # fitted OK

s.lmer.13 = lmer(log(MFD) ~ audio.cond*group+(1+group|subj)+(1+group|unique.sub.id), data=mfd.df_sub) 

s.lmer.14 = lmer(log(MFD) ~ audio.cond*group+(1+audio.cond|subj)+(1+group|unique.sub.id), data=mfd.df_sub)

s.lmer.15 = lmer(log(MFD) ~ audio.cond*group+(1+group|subj)+(1+audio.cond|unique.sub.id), data=mfd.df_sub)


s.lmer.16 = lmer(log(MFD) ~ audio.cond*group+(1|subj)+(1+audio.cond*group|unique.sub.id), data=mfd.df_sub) 

s.lmer.17 = lmer(log(MFD) ~ audio.cond*group+(1|subj)+(1+audio.cond+group|unique.sub.id), data=mfd.df_sub) 

s.lmer.18 = lmer(log(MFD) ~ audio.cond*group+(1|subj)+(1+audio.cond|unique.sub.id), data=mfd.df_sub) 

s.lmer.19 = lmer(log(MFD) ~ audio.cond*group+(1|subj)+(1+group|unique.sub.id), data=mfd.df_sub)
```

```
anova(s.lmer.min,s.lmer.08, s.lmer.12)
```

```
## Data: mfd.df_sub
## Models:
## s.lmer.min: log(MFD) ~ audio.cond * group + (1 | subj) + (1 | unique.sub.id)
## s.lmer.08: log(MFD) ~ audio.cond * group + (1 + audio.cond | subj) + (1 | unique.sub.id)
## s.lmer.12: log(MFD) ~ audio.cond * group + (1 + audio.cond | subj) + (1 + audio.cond | unique.sub.id)
##            npar     AIC     BIC logLik deviance  Chisq Df Pr(>Chisq)    
## s.lmer.min    9 -3584.7 -3508.1 1801.3  -3602.7                         
## s.lmer.08    11 -3951.8 -3858.2 1986.9  -3973.8 371.11  2  < 2.2e-16 ***
## s.lmer.12    13 -4004.4 -3893.8 2015.2  -4030.4  56.58  2  5.174e-13 ***
## ---
## Signif. codes:  0 '***' 0.001 '**' 0.01 '*' 0.05 '.' 0.1 ' ' 1
```

```
anova(s.lmer.08, s.lmer.12) # model 12
```

```
## Data: mfd.df_sub
## Models:
## s.lmer.08: log(MFD) ~ audio.cond * group + (1 + audio.cond | subj) + (1 | unique.sub.id)
## s.lmer.12: log(MFD) ~ audio.cond * group + (1 + audio.cond | subj) + (1 + audio.cond | unique.sub.id)
##           npar     AIC     BIC logLik deviance Chisq Df Pr(>Chisq)    
## s.lmer.08   11 -3951.8 -3858.2 1986.9  -3973.8                        
## s.lmer.12   13 -4004.4 -3893.8 2015.2  -4030.4 56.58  2  5.174e-13 ***
## ---
## Signif. codes:  0 '***' 0.001 '**' 0.01 '*' 0.05 '.' 0.1 ' ' 1
```

# Final MFD Model

```
#  run PCA
summary(rePCA(s.lmer.12))
```

```
## $unique.sub.id
## Importance of components:
##                          [,1]   [,2]
## Standard deviation     0.3502 0.1472
## Proportion of Variance 0.8499 0.1501
## Cumulative Proportion  0.8499 1.0000
## 
## $subj
## Importance of components:
##                          [,1]   [,2]
## Standard deviation     0.5714 0.2622
## Proportion of Variance 0.8260 0.1740
## Cumulative Proportion  0.8260 1.0000
```

```
# model details
summary(s.lmer.12)
```

```
## Linear mixed model fit by REML. t-tests use Satterthwaite's method [
## lmerModLmerTest]
## Formula: log(MFD) ~ audio.cond * group + (1 + audio.cond | subj) + (1 +  
##     audio.cond | unique.sub.id)
##    Data: mfd.df_sub
## 
## REML criterion at convergence: -3989.2
## 
## Scaled residuals: 
##     Min      1Q  Median      3Q     Max 
## -5.7197 -0.5852 -0.0542  0.5191  6.1526 
## 
## Random effects:
##  Groups        Name          Variance Std.Dev. Corr 
##  unique.sub.id (Intercept)   0.005857 0.07653       
##                audio.condOFF 0.001312 0.03623  -0.38
##  subj          (Intercept)   0.015865 0.12596       
##                audio.condOFF 0.003772 0.06142  -0.27
##  Residual                    0.049682 0.22290       
## Number of obs: 36591, groups:  unique.sub.id, 364; subj, 156
## 
## Fixed effects:
##                          Estimate Std. Error         df t value Pr(>|t|)    
## (Intercept)              5.242710   0.020119 165.999079 260.591  < 2e-16 ***
## audio.condOFF            0.044884   0.010697 155.877086   4.196 4.55e-05 ***
## groupAUS                 0.042455   0.026614 152.988852   1.595    0.113    
## groupPL                  0.025916   0.025442 152.953136   1.019    0.310    
## audio.condOFF:groupAUS  -0.003036   0.014198 145.887282  -0.214    0.831    
## audio.condOFF:groupPL   -0.012810   0.013570 145.771005  -0.944    0.347    
## ---
## Signif. codes:  0 '***' 0.001 '**' 0.01 '*' 0.05 '.' 0.1 ' ' 1
## 
## Correlation of Fixed Effects:
##             (Intr) ad.OFF grpAUS gropPL a.OFF:A
## audi.cndOFF -0.297                             
## groupAUS    -0.726  0.214                      
## groupPL     -0.759  0.224  0.574               
## ad.cOFF:AUS  0.214 -0.729 -0.293 -0.169        
## ad.cnOFF:PL  0.223 -0.763 -0.169 -0.293  0.575
```

```
# get table of model coefficients 
tab_model(s.lmer.12,file = "MFD_sub_s.lmer.12.doc")
```

|  | log(MFD) | | |
| --- | --- | --- | --- |
| Predictors | Estimates | CI | p |
| (Intercept) | 5.24 | 5.20 – 5.28 | **<0.001** |
| audio cond [OFF] | 0.04 | 0.02 – 0.07 | **<0.001** |
| group [AUS] | 0.04 | -0.01 – 0.09 | 0.111 |
| group [PL] | 0.03 | -0.02 – 0.08 | 0.308 |
| audio cond [OFF] × group [AUS] | -0.00 | -0.03 – 0.02 | 0.831 |
| audio cond [OFF] × group [PL] | -0.01 | -0.04 – 0.01 | 0.345 |
| Random Effects | | | |
| σ^2^ | 0.05 | | |
| τ_00_ _unique.sub.id_ | 0.01 | | || τ_00_ _subj_ | 0.02 | | || τ_11_ _unique.sub.id.audio.condOFF_ | 0.00 | | || τ_11_ _subj.audio.condOFF_ | 0.00 | | || ρ_01_ _unique.sub.id_ | -0.38 | | || ρ_01_ _subj_ | -0.27 | | || ICC | 0.30 | | || N _subj_ | 156 | | || N _unique.sub.id_ | 364 | | || Observations | 36591 | | |
| Marginal R^2^ / Conditional R^2^ | 0.009 / 0.304 | | |

## Extract pairwise comparisons of means

```
# Obtain marginal means of the predictions
library(emmeans)
emm <- emmeans(s.lmer.12, ~ audio.cond | group)
```

```
# plot marginal means
plot(emm)
```

```
emmip(emm, group ~ audio.cond, CIs = TRUE)
```

```
emm1 = emmeans(s.lmer.12, specs = pairwise ~ audio.cond:group)
emm1
```

```
## $emmeans
##  audio.cond group emmean     SE  df asymp.LCL asymp.UCL
##  ON         UK      5.24 0.0201 Inf      5.20      5.28
##  OFF        UK      5.29 0.0198 Inf      5.25      5.33
##  ON         AUS     5.29 0.0183 Inf      5.25      5.32
##  OFF        AUS     5.33 0.0180 Inf      5.29      5.36
##  ON         PL      5.27 0.0166 Inf      5.24      5.30
##  OFF        PL      5.30 0.0163 Inf      5.27      5.33
## 
## Degrees-of-freedom method: asymptotic 
## Results are given on the log (not the response) scale. 
## Confidence level used: 0.95 
## 
## $contrasts
##  contrast         estimate      SE  df z.ratio p.value
##  ON UK - OFF UK   -0.04488 0.01070 Inf  -4.196  0.0004
##  ON UK - ON AUS   -0.04245 0.02661 Inf  -1.595  0.6017
##  ON UK - OFF AUS  -0.08430 0.02651 Inf  -3.179  0.0185
##  ON UK - ON PL    -0.02592 0.02544 Inf  -1.019  0.9119
##  ON UK - OFF PL   -0.05799 0.02537 Inf  -2.286  0.1998
##  OFF UK - ON AUS   0.00243 0.02647 Inf   0.092  1.0000
##  OFF UK - OFF AUS -0.03942 0.02623 Inf  -1.503  0.6626
##  OFF UK - ON PL    0.01897 0.02529 Inf   0.750  0.9755
##  OFF UK - OFF PL  -0.01311 0.02508 Inf  -0.523  0.9953
##  ON AUS - OFF AUS -0.04185 0.00972 Inf  -4.306  0.0002
##  ON AUS - ON PL    0.01654 0.02405 Inf   0.688  0.9834
##  ON AUS - OFF PL  -0.01554 0.02398 Inf  -0.648  0.9873
##  OFF AUS - ON PL   0.05839 0.02394 Inf   2.439  0.1429
##  OFF AUS - OFF PL  0.02631 0.02371 Inf   1.110  0.8776
##  ON PL - OFF PL   -0.03207 0.00877 Inf  -3.656  0.0035
## 
## Degrees-of-freedom method: asymptotic 
## Results are given on the log (not the response) scale. 
## P value adjustment: tukey method for comparing a family of 6 estimates
```

```
emm1$contrasts %>%
     summary(infer = TRUE)
```

```
##  contrast         estimate      SE  df asymp.LCL asymp.UCL z.ratio p.value
##  ON UK - OFF UK   -0.04488 0.01070 Inf  -0.07537  -0.01440  -4.196  0.0004
##  ON UK - ON AUS   -0.04245 0.02661 Inf  -0.11830   0.03339  -1.595  0.6017
##  ON UK - OFF AUS  -0.08430 0.02651 Inf  -0.15986  -0.00874  -3.179  0.0185
##  ON UK - ON PL    -0.02592 0.02544 Inf  -0.09842   0.04659  -1.019  0.9119
##  ON UK - OFF PL   -0.05799 0.02537 Inf  -0.13030   0.01431  -2.286  0.1998
##  OFF UK - ON AUS   0.00243 0.02647 Inf  -0.07300   0.07786   0.092  1.0000
##  OFF UK - OFF AUS -0.03942 0.02623 Inf  -0.11418   0.03534  -1.503  0.6626
##  OFF UK - ON PL    0.01897 0.02529 Inf  -0.05311   0.09104   0.750  0.9755
##  OFF UK - OFF PL  -0.01311 0.02508 Inf  -0.08457   0.05836  -0.523  0.9953
##  ON AUS - OFF AUS -0.04185 0.00972 Inf  -0.06954  -0.01416  -4.306  0.0002
##  ON AUS - ON PL    0.01654 0.02405 Inf  -0.05199   0.08507   0.688  0.9834
##  ON AUS - OFF PL  -0.01554 0.02398 Inf  -0.08386   0.05279  -0.648  0.9873
##  OFF AUS - ON PL   0.05839 0.02394 Inf  -0.00983   0.12660   2.439  0.1429
##  OFF AUS - OFF PL  0.02631 0.02371 Inf  -0.04126   0.09389   1.110  0.8776
##  ON PL - OFF PL   -0.03207 0.00877 Inf  -0.05708  -0.00707  -3.656  0.0035
## 
## Degrees-of-freedom method: asymptotic 
## Results are given on the log (not the response) scale. 
## Confidence level used: 0.95 
## Conf-level adjustment: tukey method for comparing a family of 6 estimates 
## P value adjustment: tukey method for comparing a family of 6 estimates
```

## Covariates addition

```
glimpse(mfd.df_sub) 
mfd.df_sub$gender  = as.factor(mfd.df_sub$gender)
mfd.df_sub$AVT_Pref_bin  = as.factor(mfd.df_sub$AVT_Pref_bin)

levels(mfd.df_sub$gender)
levels(mfd.df_sub$AVT_Pref_bin)
```

```
# fit model
s.lmer.12.extra = lmer(log(MFD) ~ audio.cond*group + gender + age + education + AVT_Pref_bin + (1+audio.cond|subj)+ (1+audio.cond|unique.sub.id), data=mfd.df_sub)

summary(s.lmer.12.extra) # t-values are very small, none of the covariates are significant
```

```
## Linear mixed model fit by REML. t-tests use Satterthwaite's method [
## lmerModLmerTest]
## Formula: 
## log(MFD) ~ audio.cond * group + gender + age + education + AVT_Pref_bin +  
##     (1 + audio.cond | subj) + (1 + audio.cond | unique.sub.id)
##    Data: mfd.df_sub
## 
## REML criterion at convergence: -3959.9
## 
## Scaled residuals: 
##     Min      1Q  Median      3Q     Max 
## -5.7198 -0.5859 -0.0544  0.5192  6.1524 
## 
## Random effects:
##  Groups        Name          Variance Std.Dev. Corr 
##  unique.sub.id (Intercept)   0.005858 0.07654       
##                audio.condOFF 0.001313 0.03623  -0.38
##  subj          (Intercept)   0.015811 0.12574       
##                audio.condOFF 0.003771 0.06140  -0.28
##  Residual                    0.049682 0.22290       
## Number of obs: 36591, groups:  unique.sub.id, 364; subj, 156
## 
## Fixed effects:
##                          Estimate Std. Error         df t value Pr(>|t|)    
## (Intercept)             5.352e+00  7.100e-02  1.508e+02  75.372  < 2e-16 ***
## audio.condOFF           4.487e-02  1.070e-02  1.559e+02   4.195 4.56e-05 ***
## groupAUS                1.515e-02  2.908e-02  1.520e+02   0.521   0.6031    
## groupPL                 3.049e-02  2.616e-02  1.496e+02   1.166   0.2456    
## genderM                -3.023e-03  2.395e-02  1.480e+02  -0.126   0.8997    
## genderNB               -5.042e-02  5.779e-02  1.481e+02  -0.872   0.3844    
## age                    -2.844e-03  1.712e-03  1.480e+02  -1.661   0.0988 .  
## education              -9.366e-04  4.288e-03  1.480e+02  -0.218   0.8274    
## AVT_Pref_binsubs       -1.371e-02  3.097e-02  1.480e+02  -0.443   0.6586    
## audio.condOFF:groupAUS -3.018e-03  1.420e-02  1.459e+02  -0.213   0.8319    
## audio.condOFF:groupPL  -1.279e-02  1.357e-02  1.458e+02  -0.943   0.3473    
## ---
## Signif. codes:  0 '***' 0.001 '**' 0.01 '*' 0.05 '.' 0.1 ' ' 1
## 
## Correlation of Fixed Effects:
##             (Intr) ad.OFF grpAUS gropPL gendrM gndrNB age    eductn AVT_P_
## audi.cndOFF -0.085                                                        
## groupAUS    -0.469  0.198                                                 
## groupPL     -0.233  0.220  0.508                                          
## genderM      0.033  0.001  0.009  0.001                                   
## genderNB    -0.125  0.001  0.229  0.147  0.073                            
## age         -0.062  0.000  0.216 -0.152 -0.281  0.135                     
## education   -0.667 -0.001  0.092  0.094  0.074 -0.069 -0.582              
## AVT_Prf_bns -0.515 -0.001  0.081  0.040 -0.054  0.062  0.055  0.084       
## ad.cOFF:AUS  0.061 -0.729 -0.271 -0.166  0.000 -0.001  0.000  0.001  0.000
## ad.cnOFF:PL  0.064 -0.763 -0.156 -0.288 -0.001 -0.001  0.000  0.000  0.001
##             a.OFF:A
## audi.cndOFF        
## groupAUS           
## groupPL            
## genderM            
## genderNB           
## age                
## education          
## AVT_Prf_bns        
## ad.cOFF:AUS        
## ad.cnOFF:PL  0.575
```

```
# model explains virtually no additional variance at all
tab_model(s.lmer.12.extra,file = "MFD_sub_s.lmer.12.extra.doc")
```

|  | log(MFD) | | |
| --- | --- | --- | --- |
| Predictors | Estimates | CI | p |
| (Intercept) | 5.35 | 5.21 – 5.49 | **<0.001** |
| audio cond [OFF] | 0.04 | 0.02 – 0.07 | **<0.001** |
| group [AUS] | 0.02 | -0.04 – 0.07 | 0.602 |
| group [PL] | 0.03 | -0.02 – 0.08 | 0.244 |
| gender [M] | -0.00 | -0.05 – 0.04 | 0.900 |
| gender [NB] | -0.05 | -0.16 – 0.06 | 0.383 |
| age | -0.00 | -0.01 – 0.00 | 0.097 |
| education | -0.00 | -0.01 – 0.01 | 0.827 |
| AVT Pref bin [subs] | -0.01 | -0.07 – 0.05 | 0.658 |
| audio cond [OFF] × group [AUS] | -0.00 | -0.03 – 0.02 | 0.832 |
| audio cond [OFF] × group [PL] | -0.01 | -0.04 – 0.01 | 0.346 |
| Random Effects | | | |
| σ^2^ | 0.05 | | |
| τ_00_ _unique.sub.id_ | 0.01 | | || τ_00_ _subj_ | 0.02 | | || τ_11_ _unique.sub.id.audio.condOFF_ | 0.00 | | || τ_11_ _subj.audio.condOFF_ | 0.00 | | || ρ_01_ _unique.sub.id_ | -0.38 | | || ρ_01_ _subj_ | -0.28 | | || ICC | 0.30 | | || N _subj_ | 156 | | || N _unique.sub.id_ | 364 | | || Observations | 36591 | | |
| Marginal R^2^ / Conditional R^2^ | 0.017 / 0.309 | | |
