# Supplementary material for: Watching subtitled videos with the sound off affects viewers’ comprehension, cognitive load, immersion, enjoyment, and gaze patterns: A mixed-methods eye-tracking study
Source: PLoS One. 2024 Oct 7;19(10):e0306251. doi: 10.1371/journal.pone.0306251 (PMC11458047; doi:10.1371/journal.pone.0306251)
Supplement: S1 Data — (ZIP) [file pone.0306251.s001.zip › Data analysis_Revised after peer review_Feb 2024/SKIP_revised/SKIP_global_IA_Exp2.html]

Skipping\_Subtitle-level


# Skipping\_Subtitle-level

#### VR

#### Feb 2024

```
sessionInfo() # R version 4.3.2 (2023-10-31 ucrt)

# load libraries 
library(lattice)
library(lme4)
library(lmerTest)
library(emmeans)
library(sjPlot)
library(MASS)
library(tidyverse)
library(ggplot2)
library(pastecs)
library(udpipe)
library(optimx)
library(psych)
```

```
# import df (global IA report)
global <- read.csv ("Exp2_Global_IA report_merged.csv", header=T) # 123,032 obs, 75 vars
```

**NOTES ABOUT DATA EXCLUSIONS**:

- PL42, PL47, UK19, UK44, AUS05, AUS07, AUS51 are excluded due to
  low eye-tracking data quality.
- UK10, UK18, AUS12, AUS49, AUS51, AUS56 are excluded because they
  are not native speakers of English, or because they are
  *American* English native speakers (not British or
  Australian).

## Global dataset processing

```
summary(global)
glimpse(global)
names(global)

global$subj  = as.factor(global$subj)
global$audio.cond  = as.factor(global$audio.cond)
global$sub.cond = as.factor(global$sub.cond)
global$group  = as.factor(global$group)
global$sub.id  = as.factor(global$sub.id)
global$IA_LABEL = as.factor(global$IA_LABEL)
global$unique.sub.id = as.factor(global$unique.sub.id)
glimpse(global)
```

## Data Exclusions

```
skip.df <- data.frame(global) 

skip.df = skip.df [!skip.df$subj %in% c("PL42", "PL47", "UK19", "UK44", "AUS05", "AUS07","AUS51"),] # 117,936 rows
skip.df = skip.df [!skip.df$subj %in% c("UK10", "UK18", "AUS12", "AUS49", "AUS51", "AUS56"),] # 114,296 rows
skip.df<-skip.df[skip.df$sub.cond=="congruent",] # 76,172 rows

summary(skip.df$full.skip)
```

# IA = SUB Analysis

Skipping analysis run on the subtitle region.

```
skip.df_sub<-skip.df[skip.df$IA_LABEL=="Sub_IA",] # 38,086 obs, 75 vars
```

## Skipping Descriptives & Visualisations

```
cat("\n mean", mean(skip.df_sub$full.skip, na.rm=T)) #   mean 0.03925327
cat("\n sd", sd(skip.df_sub$full.skip, na.rm=T)) #  sd 0.1941995
```

```
psych::describe(skip.df_sub$full.skip)
```

```
##    vars     n mean   sd median trimmed mad min max range skew kurtosis se
## X1    1 38086 0.04 0.19      0       0   0   0   1     1 4.74    20.52  0
```

```
summary(skip.df_sub$full.skip)
```

```
##    Min. 1st Qu.  Median    Mean 3rd Qu.    Max. 
## 0.00000 0.00000 0.00000 0.03925 0.00000 1.00000
```

```
# means by sound
skip.df_sub %>%
  group_by(audio.cond) %>%
  summarise(avg_SKIP=mean(full.skip))
```

```
## # A tibble: 2 × 2
##   audio.cond avg_SKIP
##   <fct>         <dbl>
## 1 OFF          0.0233
## 2 ON           0.0552
```

```
# means by group (language variety)
skip.df_sub %>%
  group_by(group) %>%
  summarise(avg_SKIP=mean(full.skip))
```

```
## # A tibble: 3 × 2
##   group avg_SKIP
##   <fct>    <dbl>
## 1 AUS     0.0461
## 2 PL      0.0259
## 3 UK      0.0508
```

```
# means and SDs by sound and group 
a<-aggregate(full.skip~audio.cond*group,data=skip.df_sub,mean,na.rm=TRUE)
b<-aggregate(full.skip~audio.cond*group,data=skip.df_sub,sd,na.rm=TRUE)
meansd<-merge(a,b,by=c('audio.cond','group'))
knitr::kable(meansd, digits=3, col.names=c("Audio Condition", "Language Group","Mean","SD"))
```

| Audio Condition | Language Group | Mean | SD |
| --- | --- | --- | --- |
| OFF | AUS | 0.029 | 0.167 |
| OFF | PL | 0.016 | 0.124 |
| OFF | UK | 0.028 | 0.165 |
| ON | AUS | 0.063 | 0.243 |
| ON | PL | 0.036 | 0.187 |
| ON | UK | 0.074 | 0.261 |

```
# line graph by sound and group
skip.df_sub$audio.cond<-relevel(skip.df_sub$audio.cond,"ON")    
skip.df_sub$group<-relevel(skip.df_sub$group,"UK")
ggplot(data=skip.df_sub, aes(x=audio.cond, y=full.skip, group=group, color=group))+ 
  geom_line(stat='summary', fun.y='mean') + 
  geom_point(stat='summary', fun.y='mean') +
  xlab("Audio Condition") + ylab("Skipping Rates")
```

## Contrast Setting & releveling

```
# check contrasts
contrasts(skip.df_sub$audio.cond) # dummy/treatment coding

# sound ON as the reference level
skip.df_sub$audio.cond<-relevel(skip.df_sub$audio.cond,"ON")    

# UK as the reference level (language variety group)
skip.df_sub$group<-relevel(skip.df_sub$group,"UK")
```

# Modelling for Skipping (global analysis, IA = SUB)

Intermediary steps, including those run with uncorrelated slopes, are
omitted for brevity.

```
# failed to converge or singular fits, unless marked otherwise 
s.glmer.skip.max = glmer(full.skip ~ audio.cond*group + (1+audio.cond*group|subj) + 
                           (1+audio.cond*group|unique.sub.id), data=skip.df_sub, family=binomial) 

s.glmer.skip.01 = glmer(full.skip ~ audio.cond*group + (1+audio.cond*group|subj) + 
                          (1+audio.cond+group|unique.sub.id), data=skip.df_sub, family=binomial) 

s.glmer.skip.02 = glmer(full.skip ~ audio.cond*group + (1+audio.cond+group|subj) + 
                          (1+audio.cond*group|unique.sub.id), data=skip.df_sub, family=binomial)

s.glmer.skip.03 = glmer(full.skip ~ audio.cond*group + (1+audio.cond+group|subj) + 
                          (1+audio.cond+group|unique.sub.id), data=skip.df_sub, family=binomial) 

s.glmer.skip.04 = glmer(full.skip ~ audio.cond*group + (1+audio.cond*group|subj) + 
                          (1+audio.cond|unique.sub.id), data=skip.df_sub, family=binomial) 

s.glmer.skip.05 = glmer(full.skip ~ audio.cond*group + (1+audio.cond*group|subj) + 
                          (1+group|unique.sub.id), data=skip.df_sub, family=binomial)  

s.glmer.skip.06 = glmer(full.skip ~ audio.cond*group + (1+audio.cond*group|subj) + 
                          (1|unique.sub.id), data=skip.df_sub, family=binomial)   

s.glmer.skip.07 = glmer(full.skip ~ audio.cond*group + (1+audio.cond+group|subj) + 
                          (1+audio.cond|unique.sub.id), data=skip.df_sub, family=binomial)  

s.glmer.skip.08 = glmer(full.skip ~ audio.cond*group + (1+audio.cond+group|subj) + 
                          (1+group|unique.sub.id), data=skip.df_sub, family=binomial)  

s.glmer.skip.09 = glmer(full.skip ~ audio.cond*group + (1+audio.cond+group|subj) + 
                          (1|unique.sub.id), data=skip.df_sub, family=binomial)  

s.glmer.skip.10 = glmer(full.skip ~ audio.cond*group + (1+audio.cond|subj) + 
                          (1+audio.cond*group|unique.sub.id), data=skip.df_sub, family=binomial)  

s.glmer.skip.11 = glmer(full.skip ~ audio.cond*group + (1+audio.cond|subj) + 
                          (1+audio.cond+group|unique.sub.id), data=skip.df_sub, family=binomial)   

s.glmer.skip.12 = glmer(full.skip ~ audio.cond*group + (1+audio.cond|subj) + 
                          (1+audio.cond|unique.sub.id), data=skip.df_sub, family=binomial)  

s.glmer.skip.13 = glmer(full.skip ~ audio.cond*group + (1+audio.cond|subj) + 
                          (1+group|unique.sub.id), data=skip.df_sub, family=binomial)   

s.glmer.skip.14 = glmer(full.skip ~ audio.cond*group + (1+audio.cond|subj) +
                          (1|unique.sub.id), data=skip.df_sub, family=binomial)    

s.glmer.skip.15 = glmer(full.skip ~ audio.cond*group + (1+group|subj) + 
                          (1+audio.cond*group|unique.sub.id), data=skip.df_sub, family=binomial)    

s.glmer.skip.16 = glmer(full.skip ~ audio.cond*group + (1+group|subj) + 
                          (1+audio.cond+group|unique.sub.id), data=skip.df_sub, family=binomial)     

s.glmer.skip.17 = glmer(full.skip ~ audio.cond*group + (1+group|subj) + 
                          (1+audio.cond|unique.sub.id), data=skip.df_sub, family=binomial)   

s.glmer.skip.18 = glmer(full.skip ~ audio.cond*group + (1+group|subj) + 
                          (1+group|unique.sub.id), data=skip.df_sub, family=binomial)   

s.glmer.skip.19 = glmer(full.skip ~ audio.cond*group + (1+group|subj) +
                          (1|unique.sub.id), data=skip.df_sub, family=binomial)   

s.glmer.skip.20 = glmer(full.skip ~ audio.cond*group + (1|subj) +
                          (1+audio.cond*group|unique.sub.id), data=skip.df_sub, family=binomial)   

s.glmer.skip.21 = glmer(full.skip ~ audio.cond*group + (1|subj) +
                          (1+audio.cond+group|unique.sub.id), data=skip.df_sub, family=binomial)  

s.glmer.skip.22 = glmer(full.skip ~ audio.cond*group + (1|subj) +
                          (1+group|unique.sub.id), data=skip.df_sub, family=binomial)  

s.glmer.skip.23 = glmer(full.skip ~ audio.cond*group + (1|subj) +
                          (1+audio.cond|unique.sub.id), data=skip.df_sub, family=binomial)  

s.glmer.skip.min = glmer(full.skip ~ audio.cond*group + (1|subj) + 
                           (1|unique.sub.id), data=skip.df_sub, family=binomial)  

s.glmer.skip.24 = glmer(full.skip ~ audio.cond*group + (1|subj), data=skip.df_sub, family=binomial) # fitted

s.glmer.skip.25 = glmer(full.skip ~ audio.cond*group + (1|unique.sub.id), data=skip.df_sub, family=binomial) # fitted

s.glmer.skip.26 = glmer(full.skip ~ audio.cond*group + (1|subj) + (1|unique.sub.id), data=skip.df_sub, family=binomial, control = glmerControl(optimizer ="Nelder_Mead"))  # fitted
```

```
anova (s.glmer.skip.24, s.glmer.skip.25, s.glmer.skip.26)  # mod 26
```

```
## Data: skip.df_sub
## Models:
## s.glmer.skip.24: full.skip ~ audio.cond * group + (1 | subj)
## s.glmer.skip.25: full.skip ~ audio.cond * group + (1 | unique.sub.id)
## s.glmer.skip.26: full.skip ~ audio.cond * group + (1 | subj) + (1 | unique.sub.id)
##                 npar     AIC   BIC  logLik deviance  Chisq Df Pr(>Chisq)    
## s.glmer.skip.24    7 11249.1 11309 -5617.6  11235.1                         
## s.glmer.skip.25    7 11090.4 11150 -5538.2  11076.4  158.7  0               
## s.glmer.skip.26    8  9975.1 10044 -4979.5   9959.1 1117.3  1  < 2.2e-16 ***
## ---
## Signif. codes:  0 '***' 0.001 '**' 0.01 '*' 0.05 '.' 0.1 ' ' 1
```

## Final skipping model

```
# model details
summary(s.glmer.skip.26)
```

```
## Generalized linear mixed model fit by maximum likelihood (Laplace
##   Approximation) [glmerMod]
##  Family: binomial  ( logit )
## Formula: full.skip ~ audio.cond * group + (1 | subj) + (1 | unique.sub.id)
##    Data: skip.df_sub
## Control: glmerControl(optimizer = "Nelder_Mead")
## 
##      AIC      BIC   logLik deviance df.resid 
##   9975.1  10043.5  -4979.5   9959.1    38078 
## 
## Scaled residuals: 
##     Min      1Q  Median      3Q     Max 
## -3.2785 -0.1752 -0.1043 -0.0624 25.5223 
## 
## Random effects:
##  Groups        Name        Variance Std.Dev.
##  unique.sub.id (Intercept) 1.375    1.173   
##  subj          (Intercept) 1.259    1.122   
## Number of obs: 38086, groups:  unique.sub.id, 364; subj, 156
## 
## Fixed effects:
##                        Estimate Std. Error z value Pr(>|z|)    
## (Intercept)            -3.54914    0.19930 -17.808  < 2e-16 ***
## audio.condOFF          -1.19266    0.10775 -11.069  < 2e-16 ***
## groupAUS               -0.08445    0.25224  -0.335 0.737788    
## groupPL                -0.90793    0.24730  -3.671 0.000241 ***
## audio.condOFF:groupAUS  0.19778    0.14397   1.374 0.169514    
## audio.condOFF:groupPL   0.19995    0.15648   1.278 0.201321    
## ---
## Signif. codes:  0 '***' 0.001 '**' 0.01 '*' 0.05 '.' 0.1 ' ' 1
## 
## Correlation of Fixed Effects:
##             (Intr) ad.OFF grpAUS gropPL a.OFF:A
## audi.cndOFF -0.148                             
## groupAUS    -0.695  0.122                      
## groupPL     -0.703  0.127  0.559               
## ad.cOFF:AUS  0.118 -0.746 -0.171 -0.094        
## ad.cnOFF:PL  0.107 -0.685 -0.084 -0.189  0.514
```

```
# get table of model coefficients 
tab_model(s.glmer.skip.26, file = "sub_s.glmer.skip.26.doc")
```

|  | full.skip | | |
| --- | --- | --- | --- |
| Predictors | Odds Ratios | CI | p |
| (Intercept) | 0.03 | 0.02 – 0.04 | **<0.001** |
| audio cond [OFF] | 0.30 | 0.25 – 0.37 | **<0.001** |
| group [AUS] | 0.92 | 0.56 – 1.51 | 0.738 |
| group [PL] | 0.40 | 0.25 – 0.65 | **<0.001** |
| audio cond [OFF] × group [AUS] | 1.22 | 0.92 – 1.62 | 0.170 |
| audio cond [OFF] × group [PL] | 1.22 | 0.90 – 1.66 | 0.201 |
| Random Effects | | | |
| σ^2^ | 3.29 | | |
| τ_00_ _unique.sub.id_ | 1.38 | | || τ_00_ _subj_ | 1.26 | | || ICC | 0.44 | | || N _subj_ | 156 | | || N _unique.sub.id_ | 364 | | || Observations | 38086 | | |
| Marginal R^2^ / Conditional R^2^ | 0.069 / 0.483 | | |

## Extract pairwise comparisons of means

```
# Obtain marginal means of the predictions
library(emmeans)
emm <- emmeans(s.glmer.skip.26, ~ audio.cond | group)
```

```
# plot marginal means
plot(emm)
```

```
emmip(emm, group ~ audio.cond, CIs = TRUE)
```

```
emm1 = emmeans(s.glmer.skip.26, specs = pairwise ~ audio.cond:group)
emm1
```

```
## $emmeans
##  audio.cond group emmean    SE  df asymp.LCL asymp.UCL
##  ON         UK     -3.55 0.199 Inf     -3.94     -3.16
##  OFF        UK     -4.74 0.212 Inf     -5.16     -4.33
##  ON         AUS    -3.63 0.183 Inf     -3.99     -3.28
##  OFF        AUS    -4.63 0.193 Inf     -5.01     -4.25
##  ON         PL     -4.46 0.178 Inf     -4.81     -4.11
##  OFF        PL     -5.45 0.192 Inf     -5.83     -5.07
## 
## Results are given on the logit (not the response) scale. 
## Confidence level used: 0.95 
## 
## $contrasts
##  contrast         estimate     SE  df z.ratio p.value
##  ON UK - OFF UK     1.1927 0.1078 Inf  11.069  <.0001
##  ON UK - ON AUS     0.0844 0.2522 Inf   0.335  0.9994
##  ON UK - OFF AUS    1.0793 0.2590 Inf   4.168  0.0004
##  ON UK - ON PL      0.9079 0.2473 Inf   3.671  0.0033
##  ON UK - OFF PL     1.9006 0.2574 Inf   7.383  <.0001
##  OFF UK - ON AUS   -1.1082 0.2619 Inf  -4.231  0.0003
##  OFF UK - OFF AUS  -0.1133 0.2683 Inf  -0.422  0.9983
##  OFF UK - ON PL    -0.2847 0.2570 Inf  -1.108  0.8783
##  OFF UK - OFF PL    0.7080 0.2665 Inf   2.657  0.0841
##  ON AUS - OFF AUS   0.9949 0.0959 Inf  10.375  <.0001
##  ON AUS - ON PL     0.8235 0.2346 Inf   3.511  0.0060
##  ON AUS - OFF PL    1.8162 0.2452 Inf   7.407  <.0001
##  OFF AUS - ON PL   -0.1714 0.2416 Inf  -0.709  0.9809
##  OFF AUS - OFF PL   0.8213 0.2517 Inf   3.263  0.0141
##  ON PL - OFF PL     0.9927 0.1140 Inf   8.705  <.0001
## 
## Results are given on the log odds ratio (not the response) scale. 
## P value adjustment: tukey method for comparing a family of 6 estimates
```

```
# Results are given on the log odds ratio (not the response) scale. 
emm1$contrasts %>%
     summary(infer = TRUE)
```

```
##  contrast         estimate     SE  df asymp.LCL asymp.UCL z.ratio p.value
##  ON UK - OFF UK     1.1927 0.1078 Inf    0.8856     1.500  11.069  <.0001
##  ON UK - ON AUS     0.0844 0.2522 Inf   -0.6344     0.803   0.335  0.9994
##  ON UK - OFF AUS    1.0793 0.2590 Inf    0.3413     1.817   4.168  0.0004
##  ON UK - ON PL      0.9079 0.2473 Inf    0.2032     1.613   3.671  0.0033
##  ON UK - OFF PL     1.9006 0.2574 Inf    1.1671     2.634   7.383  <.0001
##  OFF UK - ON AUS   -1.1082 0.2619 Inf   -1.8546    -0.362  -4.231  0.0003
##  OFF UK - OFF AUS  -0.1133 0.2683 Inf   -0.8778     0.651  -0.422  0.9983
##  OFF UK - ON PL    -0.2847 0.2570 Inf   -1.0170     0.448  -1.108  0.8783
##  OFF UK - OFF PL    0.7080 0.2665 Inf   -0.0514     1.467   2.657  0.0841
##  ON AUS - OFF AUS   0.9949 0.0959 Inf    0.7216     1.268  10.375  <.0001
##  ON AUS - ON PL     0.8235 0.2346 Inf    0.1551     1.492   3.511  0.0060
##  ON AUS - OFF PL    1.8162 0.2452 Inf    1.1174     2.515   7.407  <.0001
##  OFF AUS - ON PL   -0.1714 0.2416 Inf   -0.8600     0.517  -0.709  0.9809
##  OFF AUS - OFF PL   0.8213 0.2517 Inf    0.1040     1.539   3.263  0.0141
##  ON PL - OFF PL     0.9927 0.1140 Inf    0.6677     1.318   8.705  <.0001
## 
## Results are given on the log odds ratio (not the response) scale. 
## Confidence level used: 0.95 
## Conf-level adjustment: tukey method for comparing a family of 6 estimates 
## P value adjustment: tukey method for comparing a family of 6 estimates
```

## Covariates addition

```
glimpse(skip.df_sub) 
skip.df_sub$gender  = as.factor(skip.df_sub$gender)
skip.df_sub$AVT_Pref_bin  = as.factor(skip.df_sub$AVT_Pref_bin)

levels(skip.df_sub$gender)
levels(skip.df_sub$AVT_Pref_bin)
```

```
s.glmer.skip.26.extra = glmer(full.skip ~ audio.cond*group + gender + age + education + AVT_Pref_bin + (1|subj) + (1|unique.sub.id), data=skip.df_sub, family=binomial) # fails to converge

s.glmer.skip.25.extra = glmer(full.skip ~ audio.cond*group + gender + age + education + AVT_Pref_bin + (1|unique.sub.id), data=skip.df_sub, family=binomial) # fails to converge

s.glmer.skip.24.extra = glmer(full.skip ~ audio.cond*group + gender + age + education + AVT_Pref_bin + (1|subj), data=skip.df_sub, family=binomial) # fails to converge

# try with optimizer
var.optims <- allFit(s.glmer.skip.26.extra) # bobyqa : [OK]
```

```
# bobyqa
s.glmer.skip.26.extra.1 = glmer(full.skip ~ audio.cond*group + gender + age + education + AVT_Pref_bin + (1|subj) + (1|unique.sub.id), data=skip.df_sub, family=binomial, control = glmerControl(optimizer ='bobyqa')) # fitted OK
```

```
# compare models
anova(s.glmer.skip.26, s.glmer.skip.26.extra.1) # new model (mod 26.extra.1) has lower AIC but higher BIC than the original one
```

```
## Data: skip.df_sub
## Models:
## s.glmer.skip.26: full.skip ~ audio.cond * group + (1 | subj) + (1 | unique.sub.id)
## s.glmer.skip.26.extra.1: full.skip ~ audio.cond * group + gender + age + education + AVT_Pref_bin + (1 | subj) + (1 | unique.sub.id)
##                         npar    AIC   BIC  logLik deviance  Chisq Df Pr(>Chisq)
## s.glmer.skip.26            8 9975.1 10044 -4979.5   9959.1                     
## s.glmer.skip.26.extra.1   13 9969.5 10081 -4971.7   9943.5 15.613  5   0.008042
##                           
## s.glmer.skip.26           
## s.glmer.skip.26.extra.1 **
## ---
## Signif. codes:  0 '***' 0.001 '**' 0.01 '*' 0.05 '.' 0.1 ' ' 1
```

```
summary(s.glmer.skip.26.extra.1)
```

```
## Generalized linear mixed model fit by maximum likelihood (Laplace
##   Approximation) [glmerMod]
##  Family: binomial  ( logit )
## Formula: 
## full.skip ~ audio.cond * group + gender + age + education + AVT_Pref_bin +  
##     (1 | subj) + (1 | unique.sub.id)
##    Data: skip.df_sub
## Control: glmerControl(optimizer = "bobyqa")
## 
##      AIC      BIC   logLik deviance df.resid 
##   9969.5  10080.6  -4971.7   9943.5    38073 
## 
## Scaled residuals: 
##     Min      1Q  Median      3Q     Max 
## -3.2675 -0.1756 -0.1047 -0.0625 28.3268 
## 
## Random effects:
##  Groups        Name        Variance Std.Dev.
##  unique.sub.id (Intercept) 1.374    1.172   
##  subj          (Intercept) 1.110    1.053   
## Number of obs: 38086, groups:  unique.sub.id, 364; subj, 156
## 
## Fixed effects:
##                        Estimate Std. Error z value Pr(>|z|)    
## (Intercept)            -3.82625    0.67250  -5.690 1.27e-08 ***
## audio.condOFF          -1.19360    0.10775 -11.078  < 2e-16 ***
## groupAUS               -0.34194    0.26137  -1.308 0.190776    
## groupPL                -0.80439    0.24168  -3.328 0.000874 ***
## genderM                 0.09447    0.22843   0.414 0.679193    
## genderNB               -1.09458    0.55056  -1.988 0.046798 *  
## age                    -0.06197    0.01702  -3.640 0.000273 ***
## education               0.10731    0.04114   2.608 0.009096 ** 
## AVT_Pref_binsubs        0.22830    0.29743   0.768 0.442725    
## audio.condOFF:groupAUS  0.20084    0.14388   1.396 0.162742    
## audio.condOFF:groupPL   0.19875    0.15646   1.270 0.203984    
## ---
## Signif. codes:  0 '***' 0.001 '**' 0.01 '*' 0.05 '.' 0.1 ' ' 1
## 
## Correlation of Fixed Effects:
##             (Intr) ad.OFF grpAUS gropPL gendrM gndrNB age    eductn AVT_P_
## audi.cndOFF -0.042                                                        
## groupAUS    -0.459  0.120                                                 
## groupPL     -0.200  0.130  0.486                                          
## genderM      0.038  0.002  0.009  0.028                                   
## genderNB    -0.105  0.012  0.228  0.149  0.073                            
## age         -0.054  0.001  0.220 -0.162 -0.287  0.129                     
## education   -0.663  0.000  0.086  0.088  0.072 -0.080 -0.591              
## AVT_Prf_bns -0.519 -0.007  0.080  0.035 -0.045  0.048  0.023  0.101       
## ad.cOFF:AUS  0.031 -0.746 -0.164 -0.095 -0.001 -0.007  0.000  0.000  0.008
## ad.cnOFF:PL  0.035 -0.684 -0.082 -0.194 -0.005 -0.004  0.005 -0.006  0.001
##             a.OFF:A
## audi.cndOFF        
## groupAUS           
## groupPL            
## genderM            
## genderNB           
## age                
## education          
## AVT_Prf_bns        
## ad.cOFF:AUS        
## ad.cnOFF:PL  0.513
```

```
tab_model(s.glmer.skip.26.extra.1,file = "sub_s.glmer.skip.26.extra1.doc")
```

|  | full.skip | | |
| --- | --- | --- | --- |
| Predictors | Odds Ratios | CI | p |
| (Intercept) | 0.02 | 0.01 – 0.08 | **<0.001** |
| audio cond [OFF] | 0.30 | 0.25 – 0.37 | **<0.001** |
| group [AUS] | 0.71 | 0.43 – 1.19 | 0.191 |
| group [PL] | 0.45 | 0.28 – 0.72 | **0.001** |
| gender [M] | 1.10 | 0.70 – 1.72 | 0.679 |
| gender [NB] | 0.33 | 0.11 – 0.98 | **0.047** |
| age | 0.94 | 0.91 – 0.97 | **<0.001** |
| education | 1.11 | 1.03 – 1.21 | **0.009** |
| AVT Pref bin [subs] | 1.26 | 0.70 – 2.25 | 0.443 |
| audio cond [OFF] × group [AUS] | 1.22 | 0.92 – 1.62 | 0.163 |
| audio cond [OFF] × group [PL] | 1.22 | 0.90 – 1.66 | 0.204 |
| Random Effects | | | |
| σ^2^ | 3.29 | | |
| τ_00_ _unique.sub.id_ | 1.37 | | || τ_00_ _subj_ | 1.11 | | || ICC | 0.43 | | || N _subj_ | 156 | | || N _unique.sub.id_ | 364 | | || Observations | 38086 | | |
| Marginal R^2^ / Conditional R^2^ | 0.093 / 0.483 | | |

## Extract pairwise comparisons of means (model with additional covariates)

```
# Obtain marginal means of the predictions
library(emmeans)
emm.full <- emmeans(s.glmer.skip.26.extra.1, ~ audio.cond | group)
```

```
# plot marginal means
plot(emm.full)
```

```
emmip(emm.full, group ~ audio.cond, CIs = TRUE)
```

```
emm.full.1 = emmeans(s.glmer.skip.26.extra.1, specs = pairwise ~ audio.cond:group)
emm.full.1
```

```
## $emmeans
##  audio.cond group emmean    SE  df asymp.LCL asymp.UCL
##  ON         UK     -3.92 0.263 Inf     -4.43     -3.40
##  OFF        UK     -5.11 0.274 Inf     -5.65     -4.57
##  ON         AUS    -4.26 0.289 Inf     -4.83     -3.69
##  OFF        AUS    -5.25 0.296 Inf     -5.83     -4.67
##  ON         PL     -4.72 0.270 Inf     -5.25     -4.19
##  OFF        PL     -5.72 0.281 Inf     -6.27     -5.16
## 
## Results are averaged over the levels of: gender, AVT_Pref_bin 
## Results are given on the logit (not the response) scale. 
## Confidence level used: 0.95 
## 
## $contrasts
##  contrast         estimate     SE  df z.ratio p.value
##  ON UK - OFF UK      1.194 0.1077 Inf  11.078  <.0001
##  ON UK - ON AUS      0.342 0.2614 Inf   1.308  0.7806
##  ON UK - OFF AUS     1.335 0.2681 Inf   4.979  <.0001
##  ON UK - ON PL       0.804 0.2417 Inf   3.328  0.0113
##  ON UK - OFF PL      1.799 0.2520 Inf   7.139  <.0001
##  OFF UK - ON AUS    -0.852 0.2705 Inf  -3.148  0.0204
##  OFF UK - OFF AUS    0.141 0.2768 Inf   0.510  0.9958
##  OFF UK - ON PL     -0.389 0.2515 Inf  -1.547  0.6334
##  OFF UK - OFF PL     0.606 0.2612 Inf   2.319  0.1864
##  ON AUS - OFF AUS    0.993 0.0959 Inf  10.357  <.0001
##  ON AUS - ON PL      0.462 0.2556 Inf   1.809  0.4598
##  ON AUS - OFF PL     1.457 0.2654 Inf   5.492  <.0001
##  OFF AUS - ON PL    -0.530 0.2622 Inf  -2.022  0.3295
##  OFF AUS - OFF PL    0.465 0.2715 Inf   1.711  0.5243
##  ON PL - OFF PL      0.995 0.1141 Inf   8.716  <.0001
## 
## Results are averaged over the levels of: gender, AVT_Pref_bin 
## Results are given on the log odds ratio (not the response) scale. 
## P value adjustment: tukey method for comparing a family of 6 estimates
```

```
# Results are given on the log odds ratio (not the response) scale. 
emm.full.1$contrasts %>%
     summary(infer = TRUE)
```

```
##  contrast         estimate     SE  df asymp.LCL asymp.UCL z.ratio p.value
##  ON UK - OFF UK      1.194 0.1077 Inf     0.887    1.5006  11.078  <.0001
##  ON UK - ON AUS      0.342 0.2614 Inf    -0.403    1.0868   1.308  0.7806
##  ON UK - OFF AUS     1.335 0.2681 Inf     0.571    2.0986   4.979  <.0001
##  ON UK - ON PL       0.804 0.2417 Inf     0.116    1.4931   3.328  0.0113
##  ON UK - OFF PL      1.799 0.2520 Inf     1.081    2.5175   7.139  <.0001
##  OFF UK - ON AUS    -0.852 0.2705 Inf    -1.623   -0.0807  -3.148  0.0204
##  OFF UK - OFF AUS    0.141 0.2768 Inf    -0.648    0.9300   0.510  0.9958
##  OFF UK - ON PL     -0.389 0.2515 Inf    -1.106    0.3276  -1.547  0.6334
##  OFF UK - OFF PL     0.606 0.2612 Inf    -0.139    1.3500   2.319  0.1864
##  ON AUS - OFF AUS    0.993 0.0959 Inf     0.720    1.2659  10.357  <.0001
##  ON AUS - ON PL      0.462 0.2556 Inf    -0.266    1.1909   1.809  0.4598
##  ON AUS - OFF PL     1.457 0.2654 Inf     0.701    2.2135   5.492  <.0001
##  OFF AUS - ON PL    -0.530 0.2622 Inf    -1.278    0.2170  -2.022  0.3295
##  OFF AUS - OFF PL    0.465 0.2715 Inf    -0.309    1.2381   1.711  0.5243
##  ON PL - OFF PL      0.995 0.1141 Inf     0.670    1.3201   8.716  <.0001
## 
## Results are averaged over the levels of: gender, AVT_Pref_bin 
## Results are given on the log odds ratio (not the response) scale. 
## Confidence level used: 0.95 
## Conf-level adjustment: tukey method for comparing a family of 6 estimates 
## P value adjustment: tukey method for comparing a family of 6 estimates
```
