# Supplementary material for: Watching subtitled videos with the sound off affects viewers’ comprehension, cognitive load, immersion, enjoyment, and gaze patterns: A mixed-methods eye-tracking study
Source: PLoS One. 2024 Oct 7;19(10):e0306251. doi: 10.1371/journal.pone.0306251 (PMC11458047; doi:10.1371/journal.pone.0306251)
Supplement: S1 Data — (ZIP) [file pone.0306251.s001.zip › Data analysis_Revised after peer review_Feb 2024/TRT_revised/TT_global_IA_Exp2.html]

Total Reading Times\_Subtitle-level


# Total Reading Times\_Subtitle-level

#### VR

#### Feb 2024

```
sessionInfo() # R version 4.3.2 (2023-10-31 ucrt)

# load libraries
library(lattice)
library(lme4)
library(lmerTest)
library(emmeans)
library(sjPlot)
library(MASS)
library(tidyverse)
library(ggplot2)
library(pastecs)
library(udpipe)
library(optimx)
library(psych)
library(nlme)
```

```
global <- read.csv ("Exp2_Global_IA report_merged.csv", header=T) # 123,032 obs, 75 vars
```

**NOTES ABOUT DATA EXCLUSIONS**:

- PL42, PL47, UK19, UK44, AUS05, AUS07, AUS51 are excluded due to
  low eye-tracking data quality.
- UK10, UK18, AUS12, AUS49, AUS51, AUS56 are excluded because they
  are not native speakers of English, or because they are
  *American* English native speakers (not British or
  Australian).

## Global dataset processing

```
summary(global)
glimpse(global)
names(global)
names(global)[17] <- "tt"

global$subj  = as.factor(global$subj)
global$audio.cond  = as.factor(global$audio.cond)
global$sub.cond = as.factor(global$sub.cond)
global$group  = as.factor(global$group)
global$IA_LABEL = as.factor(global$IA_LABEL)
global$sub.id  = as.factor(global$sub.id)
global$unique.sub.id = as.factor(global$unique.sub.id)
```

## Data Exclusions

```
tt.df <- data.frame(global)

tt.df = tt.df [!tt.df$subj %in% c("PL42", "PL47", "UK19", "UK44", "AUS05", "AUS07","AUS51"),] #  117,936 rows
tt.df = tt.df [!tt.df$subj %in% c("UK10", "UK18", "AUS12", "AUS49","AUS51", "AUS56"),] # 114,296 rows
tt.df<-tt.df[tt.df$sub.cond=="congruent",] # 76,172 rows

summary(tt.df$tt) 
nrow(tt.df[tt.df$tt == 0, ])  # 5822 rows
tt.df = tt.df[tt.df$tt!=0,] # 70,350 rows
summary(tt.df$tt)
```

# IA = SUB Analysis

TT analysis run on the subtitle region.

```
tt.df_sub<-tt.df[tt.df$IA_LABEL=="Sub_IA",] # 36,591 obs, 75 vars
glimpse(tt.df_sub)
```

## Total Times (TT) Descriptives & Visualisations

```
cat("\n mean", mean(tt.df_sub$tt, na.rm=T)) # mean 1126.612
cat("\n sd", sd(tt.df_sub$t, na.rm=T)) # sd 742.808
```

```
# normality plots
qqnorm(tt.df_sub$tt, main = 'Normal Q-Q plot (Total Times - Subtitle Area, TT >0)')
```

```
hist(tt.df_sub$tt, main = 'Total Times - Subtitle Area, TT > 0')
```

```
hist(log(tt.df_sub$tt), main = 'Total Times(log) - Subtitle Area, TT > 0')
```

```
hist(sqrt(tt.df_sub$tt), main = 'Total Times(sqrt) - Subtitle Area, TT > 0')
```

```
psych::describe(tt.df_sub$tt)
```

```
##    vars     n    mean     sd median trimmed   mad min  max range skew kurtosis
## X1    1 36591 1126.61 742.81    971 1047.52 732.4  61 4927  4866 0.94     0.63
##      se
## X1 3.88
```

```
# means by sound
with(tt.df_sub,tapply(tt,list(audio.cond),mean))
```

```
##       OFF        ON 
## 1252.5258  996.4642
```

```
# means by language variety (group)
with(tt.df_sub,tapply(tt,list(group),mean))
```

```
##      AUS       PL       UK 
## 1029.671 1257.226 1046.493
```

```
# means & SD by sound and language
a<-aggregate(tt~audio.cond*group,data=tt.df_sub,mean,na.rm=TRUE)
b<-aggregate(tt~audio.cond*group,data=tt.df_sub,sd,na.rm=TRUE)
meansd<-merge(a,b,by=c('audio.cond','group'))
knitr::kable(meansd, digits=1, col.names=c("Audio Condition","Language","Mean","SD"))
```

| Audio Condition | Language | Mean | SD |
| --- | --- | --- | --- |
| OFF | AUS | 1167.0 | 776.7 |
| OFF | PL | 1379.4 | 796.4 |
| OFF | UK | 1165.5 | 731.3 |
| ON | AUS | 887.7 | 623.1 |
| ON | PL | 1132.1 | 724.1 |
| ON | UK | 921.6 | 632.7 |

```
# line graph by sound and language
tt.df_sub$group<-relevel(tt.df_sub$group,"UK")
tt.df_sub$audio.cond<-relevel(tt.df_sub$audio.cond,"ON")    
ggplot(data=tt.df_sub, aes(x=audio.cond, y=tt, group=group, color=group))+ 
  geom_line(stat= 'summary', fun.y='mean') + 
  geom_point(stat='summary', fun.y='mean') +
  xlab("Audio Condition") + ylab("Total Times")
```

## Contrast Setting & releveling

```
# check contrasts
contrasts(tt.df_sub$audio.cond) # dummy/treatment coding

# sound ON as the reference level
tt.df_sub$audio.cond<-relevel(tt.df_sub$audio.cond,"ON")    

# UK as the reference level for language variety
tt.df_sub$group<-relevel(tt.df_sub$group,"UK")
```

# Modelling for TT (global analysis, IA = SUB, TT>0)

Intermediary steps, including those run with uncorrelated slopes, are
omitted for brevity.

```
# failed to converge or singular fits 
s.lmer.01 = lmer(tt ~ audio.cond*group + (1 + audio.cond*group|subj) + (1+audio.cond*group|unique.sub.id), data=tt.df_sub) 
s.lmer.02 = lmer(tt ~ audio.cond*group +  (1+audio.cond+group|subj) + (1+audio.cond*group | unique.sub.id), data=tt.df_sub) 

s.lmer.03 = lmer(tt ~ audio.cond*group +  (1+audio.cond*group|subj) + (1+audio.cond+group | unique.sub.id), data=tt.df_sub) 

s.lmer.04 = lmer(tt ~ audio.cond*group +  (1+audio.cond+group|subj) + (1+audio.cond+group | unique.sub.id), data=tt.df_sub)

s.lmer.05 = lmer(tt ~ audio.cond*group +  (1+audio.cond+group|subj) + (1+audio.cond | unique.sub.id), data=tt.df_sub)

s.lmer.06 = lmer(tt ~ audio.cond*group +  (1+audio.cond+group|subj) + (1+group | unique.sub.id), data=tt.df_sub)

s.lmer.07 = lmer(tt ~ audio.cond*group +  (1+group|subj) + (1+group | unique.sub.id), data=tt.df_sub)

s.lmer.15 = lmer(tt ~ audio.cond*group + (1+audio.cond|subj) + (1+group|unique.sub.id), data=tt.df_sub) 

s.lmer.16 = lmer(tt ~ audio.cond*group + (1+audio.cond|subj) + (1+audio.cond|unique.sub.id), data=tt.df_sub) # fitted

s.lmer.17 = lmer(tt ~ audio.cond*group + (1+audio.cond|subj) + (1|unique.sub.id), data=tt.df_sub) # fitted 

s.lmer.18 = lmer(tt ~ audio.cond*group + (1|subj) + (1|unique.sub.id), data=tt.df_sub) # fitted
```

```
anova (s.lmer.18, s.lmer.17, s.lmer.16)
```

```
## refitting model(s) with ML (instead of REML)
```

```
## Data: tt.df_sub
## Models:
## s.lmer.18: tt ~ audio.cond * group + (1 | subj) + (1 | unique.sub.id)
## s.lmer.17: tt ~ audio.cond * group + (1 + audio.cond | subj) + (1 | unique.sub.id)
## s.lmer.16: tt ~ audio.cond * group + (1 + audio.cond | subj) + (1 + audio.cond | unique.sub.id)
##           npar    AIC    BIC  logLik deviance   Chisq Df Pr(>Chisq)    
## s.lmer.18    9 543073 543150 -271528   543055                          
## s.lmer.17   11 541656 541749 -270817   541634 1421.40  2  < 2.2e-16 ***
## s.lmer.16   13 540716 540826 -270345   540690  943.81  2  < 2.2e-16 ***
## ---
## Signif. codes:  0 '***' 0.001 '**' 0.01 '*' 0.05 '.' 0.1 ' ' 1
```

```
anova(s.lmer.16,s.lmer.17) # model 16
```

```
## refitting model(s) with ML (instead of REML)
```

```
## Data: tt.df_sub
## Models:
## s.lmer.17: tt ~ audio.cond * group + (1 + audio.cond | subj) + (1 | unique.sub.id)
## s.lmer.16: tt ~ audio.cond * group + (1 + audio.cond | subj) + (1 + audio.cond | unique.sub.id)
##           npar    AIC    BIC  logLik deviance  Chisq Df Pr(>Chisq)    
## s.lmer.17   11 541656 541749 -270817   541634                         
## s.lmer.16   13 540716 540826 -270345   540690 943.81  2  < 2.2e-16 ***
## ---
## Signif. codes:  0 '***' 0.001 '**' 0.01 '*' 0.05 '.' 0.1 ' ' 1
```

# Final TT Model

```
#  run PCA:
summary(rePCA(s.lmer.16))
```

```
## $unique.sub.id
## Importance of components:
##                          [,1]    [,2]
## Standard deviation     1.4445 0.33453
## Proportion of Variance 0.9491 0.05091
## Cumulative Proportion  0.9491 1.00000
## 
## $subj
## Importance of components:
##                          [,1]   [,2]
## Standard deviation     0.5852 0.4536
## Proportion of Variance 0.6247 0.3753
## Cumulative Proportion  0.6247 1.0000
```

```
# model details
summary(s.lmer.16)
```

```
## Linear mixed model fit by REML. t-tests use Satterthwaite's method [
## lmerModLmerTest]
## Formula: tt ~ audio.cond * group + (1 + audio.cond | subj) + (1 + audio.cond |  
##     unique.sub.id)
##    Data: tt.df_sub
## 
## REML criterion at convergence: 540637.6
## 
## Scaled residuals: 
##     Min      1Q  Median      3Q     Max 
## -8.8028 -0.5733 -0.0367  0.5407  5.7249 
## 
## Random effects:
##  Groups        Name          Variance Std.Dev. Corr 
##  unique.sub.id (Intercept)   283687   532.6         
##                audio.condOFF  22035   148.4    0.53 
##  subj          (Intercept)    44764   211.6         
##                audio.condOFF  31473   177.4    -0.18
##  Residual                    139067   372.9         
## Number of obs: 36591, groups:  unique.sub.id, 364; subj, 156
## 
## Fixed effects:
##                        Estimate Std. Error     df t value Pr(>|t|)    
## (Intercept)              883.20      43.31 367.22  20.391  < 2e-16 ***
## audio.condOFF            277.48      29.48 173.25   9.413  < 2e-16 ***
## groupAUS                 -22.80      44.70 152.04  -0.510    0.611    
## groupPL                  233.68      42.73 152.00   5.469 1.82e-07 ***
## audio.condOFF:groupAUS    18.02      38.38 150.14   0.469    0.639    
## audio.condOFF:groupPL    -30.29      36.68 150.07  -0.826    0.410    
## ---
## Signif. codes:  0 '***' 0.001 '**' 0.01 '*' 0.05 '.' 0.1 ' ' 1
## 
## Correlation of Fixed Effects:
##             (Intr) ad.OFF grpAUS gropPL a.OFF:A
## audi.cndOFF -0.061                             
## groupAUS    -0.566  0.146                      
## groupPL     -0.592  0.153  0.574               
## ad.cOFF:AUS  0.116 -0.714 -0.204 -0.117        
## ad.cnOFF:PL  0.121 -0.747 -0.117 -0.204  0.574
```

```
# table of model coefficients 
tab_model(s.lmer.16,file = "TT_sub_lme_s.lmer.16.doc")
```

|  | tt | | |
| --- | --- | --- | --- |
| Predictors | Estimates | CI | p |
| (Intercept) | 883.20 | 798.30 – 968.09 | **<0.001** |
| audio cond [OFF] | 277.48 | 219.70 – 335.26 | **<0.001** |
| group [AUS] | -22.80 | -110.42 – 64.81 | 0.610 |
| group [PL] | 233.68 | 149.93 – 317.44 | **<0.001** |
| audio cond [OFF] × group [AUS] | 18.02 | -57.20 – 93.24 | 0.639 |
| audio cond [OFF] × group [PL] | -30.29 | -102.19 – 41.62 | 0.409 |
| Random Effects | | | |
| σ^2^ | 139067.29 | | |
| τ_00_ _unique.sub.id_ | 283687.42 | | || τ_00_ _subj_ | 44763.54 | | || τ_11_ _unique.sub.id.audio.condOFF_ | 22035.20 | | || τ_11_ _subj.audio.condOFF_ | 31472.64 | | || ρ_01_ _unique.sub.id_ | 0.53 | | || ρ_01_ _subj_ | -0.18 | | || ICC | 0.74 | | || N _subj_ | 156 | | || N _unique.sub.id_ | 364 | | || Observations | 36591 | | |
| Marginal R^2^ / Conditional R^2^ | 0.055 / 0.752 | | |

## Extract pairwise comparisons of means

```
# Obtain marginal means of the predictions
library(emmeans)
emm <- emmeans(s.lmer.16, ~ audio.cond | group)
```

```
# plot  marginal means:
plot(emm)
```

```
emmip(emm, group ~ audio.cond, CIs = TRUE)
```

```
emm1 = emmeans(s.lmer.16, specs = pairwise ~ audio.cond:group)
emm1
```

```
## $emmeans
##  audio.cond group emmean   SE  df asymp.LCL asymp.UCL
##  ON         UK       883 43.3 Inf       798       968
##  OFF        UK      1161 50.9 Inf      1061      1260
##  ON         AUS      860 41.0 Inf       780       941
##  OFF        AUS     1156 48.2 Inf      1061      1250
##  ON         PL      1117 38.9 Inf      1041      1193
##  OFF        PL      1364 45.6 Inf      1275      1454
## 
## Degrees-of-freedom method: asymptotic 
## Confidence level used: 0.95 
## 
## $contrasts
##  contrast         estimate   SE  df z.ratio p.value
##  ON UK - OFF UK    -277.48 29.5 Inf  -9.413  <.0001
##  ON UK - ON AUS      22.80 44.7 Inf   0.510  0.9958
##  ON UK - OFF AUS   -272.69 49.1 Inf  -5.556  <.0001
##  ON UK - ON PL     -233.68 42.7 Inf  -5.469  <.0001
##  ON UK - OFF PL    -480.88 46.6 Inf -10.323  <.0001
##  OFF UK - ON AUS    300.28 49.8 Inf   6.027  <.0001
##  OFF UK - OFF AUS     4.79 52.6 Inf   0.091  1.0000
##  OFF UK - ON PL      43.80 48.1 Inf   0.911  0.9438
##  OFF UK - OFF PL   -203.40 50.3 Inf  -4.042  0.0008
##  ON AUS - OFF AUS  -295.50 26.9 Inf -10.970  <.0001
##  ON AUS - ON PL    -256.49 40.4 Inf  -6.350  <.0001
##  ON AUS - OFF PL   -503.68 44.4 Inf -11.332  <.0001
##  OFF AUS - ON PL     39.01 45.2 Inf   0.863  0.9551
##  OFF AUS - OFF PL  -208.19 47.6 Inf  -4.375  0.0002
##  ON PL - OFF PL    -247.20 24.5 Inf -10.106  <.0001
## 
## Degrees-of-freedom method: asymptotic 
## P value adjustment: tukey method for comparing a family of 6 estimates
```

```
emm1$contrasts %>%
     summary(infer = TRUE)
```

```
##  contrast         estimate   SE  df asymp.LCL asymp.UCL z.ratio p.value
##  ON UK - OFF UK    -277.48 29.5 Inf    -361.5    -193.5  -9.413  <.0001
##  ON UK - ON AUS      22.80 44.7 Inf    -104.6     150.2   0.510  0.9958
##  ON UK - OFF AUS   -272.69 49.1 Inf    -412.6    -132.8  -5.556  <.0001
##  ON UK - ON PL     -233.68 42.7 Inf    -355.5    -111.9  -5.469  <.0001
##  ON UK - OFF PL    -480.88 46.6 Inf    -613.6    -348.1 -10.323  <.0001
##  OFF UK - ON AUS    300.28 49.8 Inf     158.3     442.3   6.027  <.0001
##  OFF UK - OFF AUS     4.79 52.6 Inf    -145.2     154.8   0.091  1.0000
##  OFF UK - ON PL      43.80 48.1 Inf     -93.2     180.8   0.911  0.9438
##  OFF UK - OFF PL   -203.40 50.3 Inf    -346.8     -60.0  -4.042  0.0008
##  ON AUS - OFF AUS  -295.50 26.9 Inf    -372.3    -218.7 -10.970  <.0001
##  ON AUS - ON PL    -256.49 40.4 Inf    -371.6    -141.4  -6.350  <.0001
##  ON AUS - OFF PL   -503.68 44.4 Inf    -630.3    -377.0 -11.332  <.0001
##  OFF AUS - ON PL     39.01 45.2 Inf     -89.8     167.8   0.863  0.9551
##  OFF AUS - OFF PL  -208.19 47.6 Inf    -343.8     -72.6  -4.375  0.0002
##  ON PL - OFF PL    -247.20 24.5 Inf    -316.9    -177.5 -10.106  <.0001
## 
## Degrees-of-freedom method: asymptotic 
## Confidence level used: 0.95 
## Conf-level adjustment: tukey method for comparing a family of 6 estimates 
## P value adjustment: tukey method for comparing a family of 6 estimates
```

## Covariates addition

```
glimpse(tt.df_sub) 
tt.df_sub$gender  = as.factor(tt.df_sub$gender)
tt.df_sub$AVT_Pref_bin  = as.factor(tt.df_sub$AVT_Pref_bin)

levels(tt.df_sub$gender)
levels(tt.df_sub$AVT_Pref_bin)
```

```
# fit model
s.lmer.16.extra = lmer(tt ~ audio.cond*group + gender + age + education + AVT_Pref_bin + (1+audio.cond|subj) + (1+audio.cond|unique.sub.id), data=tt.df_sub) 

summary(s.lmer.16.extra) # t-values are very small, none of the covariates are significant
```

```
## Linear mixed model fit by REML. t-tests use Satterthwaite's method [
## lmerModLmerTest]
## Formula: tt ~ audio.cond * group + gender + age + education + AVT_Pref_bin +  
##     (1 + audio.cond | subj) + (1 + audio.cond | unique.sub.id)
##    Data: tt.df_sub
## 
## REML criterion at convergence: 540594.5
## 
## Scaled residuals: 
##     Min      1Q  Median      3Q     Max 
## -8.8028 -0.5737 -0.0369  0.5406  5.7248 
## 
## Random effects:
##  Groups        Name          Variance Std.Dev. Corr 
##  unique.sub.id (Intercept)   283641   532.6         
##                audio.condOFF  22043   148.5    0.53 
##  subj          (Intercept)    45026   212.2         
##                audio.condOFF  31487   177.4    -0.17
##  Residual                    139067   372.9         
## Number of obs: 36591, groups:  unique.sub.id, 364; subj, 156
## 
## Fixed effects:
##                        Estimate Std. Error      df t value Pr(>|t|)    
## (Intercept)             926.492    125.728 163.964   7.369 8.00e-12 ***
## audio.condOFF           277.524     29.485 173.215   9.412  < 2e-16 ***
## groupAUS                 -8.466     49.298 149.037  -0.172   0.8639    
## groupPL                 219.232     44.200 147.792   4.960 1.91e-06 ***
## genderM                 -14.632     41.504 147.684  -0.353   0.7249    
## genderNB                 14.665    100.174 147.871   0.146   0.8838    
## age                       5.369      2.968 147.685   1.809   0.0724 .  
## education                -9.836      7.433 147.808  -1.323   0.1877    
## AVT_Pref_binsubs        -21.703     53.663 147.683  -0.404   0.6865    
## audio.condOFF:groupAUS   17.944     38.385 150.103   0.467   0.6408    
## audio.condOFF:groupPL   -30.301     36.692 150.042  -0.826   0.4102    
## ---
## Signif. codes:  0 '***' 0.001 '**' 0.01 '*' 0.05 '.' 0.1 ' ' 1
## 
## Correlation of Fixed Effects:
##             (Intr) ad.OFF grpAUS gropPL gendrM gndrNB age    eductn AVT_P_
## audi.cndOFF -0.019                                                        
## groupAUS    -0.459  0.127                                                 
## groupPL     -0.223  0.142  0.504                                          
## genderM      0.032  0.001  0.010  0.002                                   
## genderNB    -0.122  0.001  0.235  0.151  0.073                            
## age         -0.060  0.001  0.221 -0.156 -0.281  0.135                     
## education   -0.653 -0.001  0.094  0.097  0.074 -0.070 -0.582              
## AVT_Prf_bns -0.503 -0.001  0.083  0.041 -0.054  0.061  0.055  0.085       
## ad.cOFF:AUS  0.038 -0.714 -0.177 -0.109  0.000 -0.001 -0.001  0.001  0.000
## ad.cnOFF:PL  0.040 -0.747 -0.102 -0.189 -0.001 -0.001  0.000  0.000  0.000
##             a.OFF:A
## audi.cndOFF        
## groupAUS           
## groupPL            
## genderM            
## genderNB           
## age                
## education          
## AVT_Prf_bns        
## ad.cOFF:AUS        
## ad.cnOFF:PL  0.574
```

```
# model explains virtually no additional variance at all
tab_model(s.lmer.16.extra,file = "TT_sub_lme_s.lmer.16.extra.doc")
```

|  | tt | | |
| --- | --- | --- | --- |
| Predictors | Estimates | CI | p |
| (Intercept) | 926.49 | 680.06 – 1172.92 | **<0.001** |
| audio cond [OFF] | 277.52 | 219.73 – 335.31 | **<0.001** |
| group [AUS] | -8.47 | -105.09 – 88.16 | 0.864 |
| group [PL] | 219.23 | 132.60 – 305.87 | **<0.001** |
| gender [M] | -14.63 | -95.98 – 66.72 | 0.724 |
| gender [NB] | 14.66 | -181.68 – 211.01 | 0.884 |
| age | 5.37 | -0.45 – 11.19 | 0.070 |
| education | -9.84 | -24.40 – 4.73 | 0.186 |
| AVT Pref bin [subs] | -21.70 | -126.88 – 83.48 | 0.686 |
| audio cond [OFF] × group [AUS] | 17.94 | -57.29 – 93.18 | 0.640 |
| audio cond [OFF] × group [PL] | -30.30 | -102.22 – 41.62 | 0.409 |
| Random Effects | | | |
| σ^2^ | 139066.93 | | |
| τ_00_ _unique.sub.id_ | 283641.47 | | || τ_00_ _subj_ | 45026.48 | | || τ_11_ _unique.sub.id.audio.condOFF_ | 22042.92 | | || τ_11_ _subj.audio.condOFF_ | 31486.61 | | || ρ_01_ _unique.sub.id_ | 0.53 | | || ρ_01_ _subj_ | -0.17 | | || ICC | 0.74 | | || N _subj_ | 156 | | || N _unique.sub.id_ | 364 | | || Observations | 36591 | | |
| Marginal R^2^ / Conditional R^2^ | 0.056 / 0.753 | | |
