# Supplementary material for: Watching subtitled videos with the sound off affects viewers’ comprehension, cognitive load, immersion, enjoyment, and gaze patterns: A mixed-methods eye-tracking study
Source: PLoS One. 2024 Oct 7;19(10):e0306251. doi: 10.1371/journal.pone.0306251 (PMC11458047; doi:10.1371/journal.pone.0306251)
Supplement: Attachment — Submitted filename: PLOS comments.docx [file pone.0306251.s002.docx]

Comments:

1. Details of questionnaire was not defined properly, was it a self-designed by the research team of the project or adopted?

Same observation holds for semi-structured interview.

NOTE: If both instruments were adopted then whether reliability and validity of both were tested in the given research context. I don’t find such information in any part of the part of the paper. If not then it must be tested and validated.

2. Descriptive data analysis result was presented in paragraph form, it better that it should be presented in tables to give comparative view between 03 data sets of 03 groups.
3. When writing results please cite the calculated figures for reference, as it gives comparative analysis with previous conducted researches in the said domain.
4. In such huge project work no theory or theoretical framework was used. Using theoretical framework will help researchers to build conceptual framework. That’s gives relationships among variables used in research.
5. As mixed method was chosen then why no mix method model was used. Please see Creswell book for the models.
6. No data triangulation was carried out in the research, as this is key component of mixed method.
7. It’s my humble request that when such research where cognition or comprehension is used, then either use educational frameworks like bloom’s taxonomy or to consult any medical doctor as a consultant, where he can brief the cognitive functions clearly.
