# Supplementary material for: Watching subtitled videos with the sound off affects viewers’ comprehension, cognitive load, immersion, enjoyment, and gaze patterns: A mixed-methods eye-tracking study
Source: PLoS One. 2024 Oct 7;19(10):e0306251. doi: 10.1371/journal.pone.0306251 (PMC11458047; doi:10.1371/journal.pone.0306251)
Supplement: S1 Data — (ZIP) [file pone.0306251.s001.zip › Data analysis_Revised after peer review_Feb 2024/CC_revised/CC_lme_cc.lmer.17.extra.doc]

|  | **cross.count** | | |
| --- | --- | --- | --- |
| *Predictors* | *Estimates* | *CI* | *p* |
| (Intercept) | 2.17 | 1.96 – 2.38 | **<0.001** |
| audio cond [OFF] | -0.06 | -0.12 – 0.01 | 0.096 |
| group [AUS] | 0.08 | -0.00 – 0.16 | 0.054 |
| group [PL] | -0.05 | -0.12 – 0.03 | 0.231 |
| gender [M] | 0.03 | -0.04 – 0.10 | 0.404 |
| gender [NB] | 0.05 | -0.11 – 0.22 | 0.518 |
| age | -0.00 | -0.01 – 0.00 | 0.231 |
| education | -0.00 | -0.02 – 0.01 | 0.584 |
| AVT Pref bin [Subs] | -0.06 | -0.15 – 0.03 | 0.216 |
| audio cond [OFF] × group [AUS] | 0.01 | -0.08 – 0.10 | 0.844 |
| audio cond [OFF] × group [PL] | -0.08 | -0.17 – 0.00 | 0.056 |
| **Random Effects** | | | |
| σ^2^ | 0.65 | | |
| τ00 unique.sub.id | 0.15 | | |
| τ00 subj | 0.03 | | |
| τ11 subj.audio.condOFF | 0.03 | | |
| ρ01 subj | -0.12 | | |
| ICC | 0.23 | | |
| N subj | 156 | | |
| N unique.sub.id | 364 | | |
| Observations | 33439 | | |
| Marginal R^2^ / Conditional R^2^ | 0.013 / 0.239 | | |
