# Supplementary material for: Watching subtitled videos with the sound off affects viewers’ comprehension, cognitive load, immersion, enjoyment, and gaze patterns: A mixed-methods eye-tracking study
Source: PLoS One. 2024 Oct 7;19(10):e0306251. doi: 10.1371/journal.pone.0306251 (PMC11458047; doi:10.1371/journal.pone.0306251)
Supplement: S1 Data — (ZIP) [file pone.0306251.s001.zip › Data analysis_Revised after peer review_Feb 2024/MFD_revised/MFD_sub_s.lmer.12.doc]

|  | **log(MFD)** | | |
| --- | --- | --- | --- |
| *Predictors* | *Estimates* | *CI* | *p* |
| (Intercept) | 5.24 | 5.20 – 5.28 | **<0.001** |
| audio cond [OFF] | 0.04 | 0.02 – 0.07 | **<0.001** |
| group [AUS] | 0.04 | -0.01 – 0.09 | 0.111 |
| group [PL] | 0.03 | -0.02 – 0.08 | 0.308 |
| audio cond [OFF] × group [AUS] | -0.00 | -0.03 – 0.02 | 0.831 |
| audio cond [OFF] × group [PL] | -0.01 | -0.04 – 0.01 | 0.345 |
| **Random Effects** | | | |
| σ^2^ | 0.05 | | |
| τ00 unique.sub.id | 0.01 | | |
| τ00 subj | 0.02 | | |
| τ11 unique.sub.id.audio.condOFF | 0.00 | | |
| τ11 subj.audio.condOFF | 0.00 | | |
| ρ01 unique.sub.id | -0.38 | | |
| ρ01 subj | -0.27 | | |
| ICC | 0.30 | | |
| N subj | 156 | | |
| N unique.sub.id | 364 | | |
| Observations | 36591 | | |
| Marginal R^2^ / Conditional R^2^ | 0.009 / 0.304 | | |
