# Supplementary material for: Watching subtitled videos with the sound off affects viewers’ comprehension, cognitive load, immersion, enjoyment, and gaze patterns: A mixed-methods eye-tracking study
Source: PLoS One. 2024 Oct 7;19(10):e0306251. doi: 10.1371/journal.pone.0306251 (PMC11458047; doi:10.1371/journal.pone.0306251)
Supplement: S1 Data — (ZIP) [file pone.0306251.s001.zip › Data analysis_Revised after peer review_Feb 2024/SKIP_revised/sub_s.glmer.skip.26.doc]

|  | **full.skip** | | |
| --- | --- | --- | --- |
| *Predictors* | *Odds Ratios* | *CI* | *p* |
| (Intercept) | 0.03 | 0.02 – 0.04 | **<0.001** |
| audio cond [OFF] | 0.30 | 0.25 – 0.37 | **<0.001** |
| group [AUS] | 0.92 | 0.56 – 1.51 | 0.738 |
| group [PL] | 0.40 | 0.25 – 0.65 | **<0.001** |
| audio cond [OFF] × group [AUS] | 1.22 | 0.92 – 1.62 | 0.169 |
| audio cond [OFF] × group [PL] | 1.22 | 0.90 – 1.66 | 0.201 |
| **Random Effects** | | | |
| σ^2^ | 3.29 | | |
| τ00 unique.sub.id | 1.38 | | |
| τ00 subj | 1.26 | | |
| ICC | 0.44 | | |
| N subj | 156 | | |
| N unique.sub.id | 364 | | |
| Observations | 38086 | | |
| Marginal R^2^ / Conditional R^2^ | 0.069 / 0.483 | | |
