# Supplementary material for: Watching subtitled videos with the sound off affects viewers’ comprehension, cognitive load, immersion, enjoyment, and gaze patterns: A mixed-methods eye-tracking study
Source: PLoS One. 2024 Oct 7;19(10):e0306251. doi: 10.1371/journal.pone.0306251 (PMC11458047; doi:10.1371/journal.pone.0306251)
Supplement: S1 Data — (ZIP) [file pone.0306251.s001.zip › Data analysis_Revised after peer review_Feb 2024/SKIP_revised/sub_s.glmer.skip.26.extra1.doc]

|  | **full.skip** | | |
| --- | --- | --- | --- |
| *Predictors* | *Odds Ratios* | *CI* | *p* |
| (Intercept) | 0.02 | 0.01 – 0.08 | **<0.001** |
| audio cond [OFF] | 0.30 | 0.25 – 0.37 | **<0.001** |
| group [AUS] | 0.71 | 0.43 – 1.19 | 0.191 |
| group [PL] | 0.45 | 0.28 – 0.72 | **0.001** |
| gender [M] | 1.10 | 0.70 – 1.72 | 0.679 |
| gender [NB] | 0.33 | 0.11 – 0.98 | **0.047** |
| age | 0.94 | 0.91 – 0.97 | **<0.001** |
| education | 1.11 | 1.03 – 1.21 | **0.009** |
| AVT Pref bin [subs] | 1.26 | 0.70 – 2.25 | 0.443 |
| audio cond [OFF] × group [AUS] | 1.22 | 0.92 – 1.62 | 0.163 |
| audio cond [OFF] × group [PL] | 1.22 | 0.90 – 1.66 | 0.204 |
| **Random Effects** | | | |
| σ^2^ | 3.29 | | |
| τ00 unique.sub.id | 1.37 | | |
| τ00 subj | 1.11 | | |
| ICC | 0.43 | | |
| N subj | 156 | | |
| N unique.sub.id | 364 | | |
| Observations | 38086 | | |
| Marginal R^2^ / Conditional R^2^ | 0.093 / 0.483 | | |
