# Supplementary material for: Watching subtitled videos with the sound off affects viewers’ comprehension, cognitive load, immersion, enjoyment, and gaze patterns: A mixed-methods eye-tracking study
Source: PLoS One. 2024 Oct 7;19(10):e0306251. doi: 10.1371/journal.pone.0306251 (PMC11458047; doi:10.1371/journal.pone.0306251)
Supplement: S1 Data — (ZIP) [file pone.0306251.s001.zip › Data analysis_Revised after peer review_Feb 2024/TRT_revised/TT_sub_lme_s.lmer.16.doc]

|  | **tt** | | |
| --- | --- | --- | --- |
| *Predictors* | *Estimates* | *CI* | *p* |
| (Intercept) | 883.20 | 798.30 – 968.10 | **<0.001** |
| audio cond [OFF] | 277.48 | 219.70 – 335.26 | **<0.001** |
| group [AUS] | -22.80 | -110.42 – 64.81 | 0.610 |
| group [PL] | 233.68 | 149.93 – 317.44 | **<0.001** |
| audio cond [OFF] × group [AUS] | 18.02 | -57.20 – 93.23 | 0.639 |
| audio cond [OFF] × group [PL] | -30.29 | -102.19 – 41.61 | 0.409 |
| **Random Effects** | | | |
| σ^2^ | 139067.11 | | |
| τ00 unique.sub.id | 283712.37 | | |
| τ00 subj | 44764.73 | | |
| τ11 unique.sub.id.audio.condOFF | 22036.90 | | |
| τ11 subj.audio.condOFF | 31470.72 | | |
| ρ01 unique.sub.id | 0.53 | | |
| ρ01 subj | -0.18 | | |
| ICC | 0.74 | | |
| N subj | 156 | | |
| N unique.sub.id | 364 | | |
| Observations | 36591 | | |
| Marginal R^2^ / Conditional R^2^ | 0.055 / 0.752 | | |
