# Supplementary material for: Watching subtitled videos with the sound off affects viewers’ comprehension, cognitive load, immersion, enjoyment, and gaze patterns: A mixed-methods eye-tracking study
Source: PLoS One. 2024 Oct 7;19(10):e0306251. doi: 10.1371/journal.pone.0306251 (PMC11458047; doi:10.1371/journal.pone.0306251)
Supplement: S1 Data — (ZIP) [file pone.0306251.s001.zip › Data analysis_Revised after peer review_Feb 2024/TRT_revised/TT_sub_lme_s.lmer.16.extra.doc]

|  | **tt** | | |
| --- | --- | --- | --- |
| *Predictors* | *Estimates* | *CI* | *p* |
| (Intercept) | 926.49 | 680.06 – 1172.92 | **<0.001** |
| audio cond [OFF] | 277.52 | 219.73 – 335.31 | **<0.001** |
| group [AUS] | -8.47 | -105.09 – 88.16 | 0.864 |
| group [PL] | 219.23 | 132.60 – 305.87 | **<0.001** |
| gender [M] | -14.63 | -95.98 – 66.72 | 0.724 |
| gender [NB] | 14.66 | -181.68 – 211.01 | 0.884 |
| age | 5.37 | -0.45 – 11.19 | 0.070 |
| education | -9.84 | -24.40 – 4.73 | 0.186 |
| AVT Pref bin [subs] | -21.70 | -126.88 – 83.48 | 0.686 |
| audio cond [OFF] × group [AUS] | 17.94 | -57.29 – 93.18 | 0.640 |
| audio cond [OFF] × group [PL] | -30.30 | -102.22 – 41.62 | 0.409 |
| **Random Effects** | | | |
| σ^2^ | 139066.93 | | |
| τ00 unique.sub.id | 283641.47 | | |
| τ00 subj | 45026.48 | | |
| τ11 unique.sub.id.audio.condOFF | 22042.92 | | |
| τ11 subj.audio.condOFF | 31486.61 | | |
| ρ01 unique.sub.id | 0.53 | | |
| ρ01 subj | -0.17 | | |
| ICC | 0.74 | | |
| N subj | 156 | | |
| N unique.sub.id | 364 | | |
| Observations | 36591 | | |
| Marginal R^2^ / Conditional R^2^ | 0.056 / 0.753 | | |
